# The effect of spatial attention on saccadic adaptation

**DOI:** 10.1167/jov.25.14.13

**Published:** 2025-12-17

**Authors:** Ali Batikh, Éric Koun, Roméo Salemme, Alessandro Farnè, Denis Pélisson

**Affiliations:** 1 IMPACT Team of Lyon Neuroscience Research Center INSERM U1028 CNRS UMR5292 University Claude Bernard Lyon 1, Lyon, France

**Keywords:** saccadic adaptation, spatial attention, cross-modal attention

## Abstract

Eye movements and spatial attention are both crucial to visual perception. Orienting gaze to objects of interest is achieved by voluntary saccades (VSs) driven by internal goals or reactive saccades (RSs) triggered automatically by sudden environmental changes. Both VSs and RSs are known to undergo plastic adjustments to maintain their accuracy throughout life, driven by saccadic adaptation processes. Spatial attention enhances visual processing within a restricted zone, and it can be shifted voluntarily following our internal goals (endogenous) or automatically in response to unexpected changes in sensory stimulation (exogenous). Despite the widely accepted notion that saccadic and attention shifts are governed by distinct but highly interconnected systems, the relationship between saccadic adaptation and spatial attention is still unclear. To address this relationship, we conducted two experiments combining modified versions of the double-step adaptation paradigm and the attention-orienting paradigm. Experiment 1 tested the effect of shifting exogenous attention by a tactile cue near or away from the saccade's target on RS adaptation. Experiment 2 also used tactile cueing but now to investigate the effect of shifting endogenous attention on VS adaptation. Although we were unable to obtain direct evidence for an effect of spatial attention on saccadic adaptation, correlation analyses indicated that both the rate and magnitude of saccadic adaptation were positively correlated with the allocation of attention toward the saccade target and negatively correlated with attention directed away from the target.

## Introduction

The fovea is restricted to 1% to 2% of the retinal surface, where visual acuity is the highest. To inspect an object of interest in our visual environment in detail, it is necessary to bring its image onto the fovea via rapid eye movements, called saccades. Saccades elicited by the sudden appearance of objects are classified as reactive (also called “reflexive” or “automatic”) saccades (RSs), and those generated based on internal goals are classified as voluntary saccades (VSs). Because of their high speed, saccades toward visual targets differ from actions involving the skeletal system in that they cannot benefit from visual feedback signals to control their execution “online” (except for large saccades [Gaveau et al., 2003] or pathologically slowed saccades [Zee, Optican, Cook, Robinson, & Engel, 1976]). Therefore, the motor programming of saccades must be optimal to ensure their accuracy despite short- to long-term modifications of the body–space relationship (growth, aging, etc.) or consequences of cerebral or neuromuscular lesions. A critical mechanism contributing to such oculomotor calibration is the so-called saccadic adaptation, a plasticity-based process that modifies saccades’ metrics to maintain their accuracy (Hopp & Fuchs, 2004; Pélisson, Alahyane, Panouillères, & Tilikete, 2010). Saccadic adaptation has been intensively studied, largely thanks to the “double-step target paradigm” (McLaughlin, 1967). In this paradigm, participants follow with their eyes a target appearing in the visual field (first step) and then changing position (second step) during the execution of the saccadic response to the first target step. Unless the second step exceeds ∼30% of the first-step amplitude, it is usually undetected due to the saccadic suppression phenomenon (Bridgeman, Hendry, & Stark, 1975; Wurtz, 2008). Nonetheless, it is interpreted by the sensorimotor system as an aiming error of the primary saccade. Repeated exposure to such aiming error in successive “double-step” trials entails the adaptation process to gradually modify the primary saccade amplitude, either reducing or increasing it, depending on the direction of the second target step relative to the first step (“backward” or “forward,” respectively), resulting in a reduction of the aiming error.

The many features within our visual field continuously and simultaneously stimulate our retinal receptors, leading to a high throughput of information transmitted to the visual system. To avoid neural processing overload, our attentional system selects salient or task-relevant information and discards nonessential information (Posner, 1980). Individuals are able to orient their attention whenever changes in the environment or the task at hand require it (Corbetta, Patel, & Shulman, 2008), calling for two types of spatial attention shifts. The first process (involuntary, “bottom-up,” or “stimulus-driven”) corresponds to the automatic capture (exogenous) of attention by salient stimuli. The second process (voluntary or “top-down”) directs attention toward stimuli defined endogenously, according to our ongoing task and current priorities (Corbetta et al., 2008; Groner & Groner, 1989). Spatial attention was studied thoroughly, either with (overt attention) or without eye movements (covert attention), thanks to different paradigms, among which is the cue–target paradigm introduced by Posner (Posner, 1980; Posner & Cohen, 1984). This paradigm consists of orienting participants’ spatial attention to a certain location using a cue stimulus that can be either central (e.g., an arrow) or peripheral (e.g., the illumination of a peripheral placeholder). After a certain delay (stimulus onset asynchrony [SOA]), participants receive a second stimulation (the target stimulus) either at the cued location (valid trials) or at the opposite location (invalid trials). Participants are usually faster and more accurate at discriminating the location or the characteristics of the target in valid trials than in invalid trials. This response speed-up in valid trials is generally attributed to a facilitation effect of the cue on target processing. In contrast, the response time cost in invalid trials is attributed to the time spatial attention takes to disengage from the cued location and shift toward the target location (Chica, Martín-Arévalo, Botta, & Lupiáñez, 2014). Spatially predictive cues (high probability of the cue indicating the correct target location, e.g., 80% valid vs. 20% invalid trials), best preceding the target by a relatively long SOA (1–2 seconds), are typically used to orient participants’ endogenous attention. In contrast, salient and nonpredictive cues presented in the periphery with shorter SOAs (<500 ms) are used to orient exogenous attention (Chica et al., 2014).

Spatial attention and eye movements are known to interact strongly. Both spatial attention and saccade preparation are reflected within the activity of the frontal eye field (FEF), the superior colliculi (SC), and the parietal eye field (Hunt, Reuther, Hilchey, & Klein, 2019). In addition, some studies have suggested the existence of a coupling between visuospatial attention and saccadic adaptation in particular. Indeed, behavioral studies in healthy participants have proposed that the adaptation of VS favors the orienting of endogenous visual attention (Nicolas, Bidet-Caulet, & Pélisson, 2019), while the adaptation of RS facilitates the orienting of exogenous attention (Habchi et al., 2015; Nicolas, Bidet-Caulet, & Pélisson, 2020). Saccadic adaptation has also been suggested to alter the attention field, expanding and shrinking in relation to forward and backward adaptation, respectively (Wick, Garaas, & Pomplun, 2016). At the neurophysiological level, Nicolas et al. (Nicolas et al., 2019) demonstrated through magnetoencephalography that adaptation of RS is accompanied by activation of an extended parietal-temporal zone overlapping with the visuospatial attention networks. Furthermore, combining the findings of functional magnetic resonance imaging (fMRI) studies of attention (Corbetta et al., 2008) and saccadic adaptation (Gerardin, Miquée, Urquizar, & Pélisson, 2012) shows that the neural substrates of exogenous spatial attention and those of RS adaptation overlap at the level of the right temporoparietal junction (rTPJ), while those of endogenous spatial attention and VS adaptation overlap at the level of the intraparietal sulcus (IPS). Taken together, these studies suggest that saccadic adaptation and visuospatial attention are functionally coupled.

Here, to further test this functional coupling hypothesis, we aimed to verify whether, in addition to the effect of adaptation on attention discussed above (Habchi et al., 2015; Nicolas et al., 2019; Nicolas et al., 2019; Nicolas et al., 2020; Wick et al., 2016), there is also an effect of attention on adaptation. Some studies have provided indirect arguments for this hypothesis, as detailed in the following. First, patients with attention-deficit/hyperactivity disorder display reduced saccadic adaptation, possibly resulting from their altered attentional capabilities (Connolly, Rinehart, & Fielding, 2016). Second, Bock, Grigorova, and Ilieva-Staneva (2017) used a scrambled sentence task to prime the participants’ attentional focus prior to RS adaptation exposure. They suggested that the postulated widening, relative to narrowing, of attentional focus increased the amount of saccadic adaptation induced during the subsequent exposure phase but not the adaptation after-effect. The same team, however, had previously found no evidence for an impact on saccadic adaptation of diverting attentional resources toward a secondary task performed concurrently (Bock, Ilieva, & Grigorova, 2014), suggesting that saccadic adaptation does not draw on spatial attention. Third, in a visually triggered saccade task, Khan et al. (Khan, McFadden, Harwood, & Wallman, 2014) presented at the time of saccade onset a salient visual distractor in the vicinity of the saccade target (±3 degrees). They found that despite the saccade target remaining stationary, the salient distractor was enough to induce saccadic adaptation, which the authors interpreted as the locus of attention drawn toward the distractor acting as an error signal for the saccadic adaptation process. In contrast, another study where either the target or the distractor jumped intra-saccadically (Madelain, Harwood, Herman, & Wallman, 2010) concluded that adaptation is induced only by target jumps and is unhampered by distractors, but note that here the distractor and target saliency were comparable. Two further studies argue for an effect of attention on saccadic adaptation (Gerardin, Nicolas, Farnè, & Pélisson, 2015; McFadden, Khan, & Wallman, 2002). In the first (McFadden et al., 2002), the authors modified the line motion illusion protocol (a static visual line presented immediately after a flashed cue is perceived as growing over time from the cue location) to determine the time at which participants’ spatial attention shifts toward the location of a peripheral target. At the time of the attentional shifts, the peripheral target stepped to another backward or forward location, leading to a progressive change in the “covert” displacement of the focus of visual attention. The authors reported that this supposedly adaptive change in covert attention led to a change in the size of RS. However, whether their procedure really adapts spatial attention remains uncertain. In the second study, Gerardin et al. (2015) tested the effect of the attentional load deployed during the adaptation exposure on RS adaptation. In every exposure trial, participants performed a double task (saccade and discrimination), as the participants had to discriminate the Gabor stimuli used as targets that jumped during the saccade to elicit adaptation. The attentional load level was varied in separate sessions as “low” or “high” by providing participants with different instructions for the discrimination task. The results showed stronger saccadic adaptation in the high-load condition (difficult discrimination) compared to the low-load condition (easy discrimination). However, this study did not allow for the disentangling of whether this effect was due to spatial attention specifically or to a general effect of alertness.

Here, we investigated more directly the effect of spatial attention on saccadic adaptation. Like most of the saccadic adaptation studies presented above, we focused on backward adaptation, as it is well known that forward adaptation is harder to induce than backward adaptation (Ethier, Zee, & Shadmehr, 2008; Panouillères et al., 2009; Pélisson et al., 2010). To more specifically manipulate spatial attention than in Gerardin et al. (2015), (a) we combined the Posner paradigm (Posner, 1980; Posner & Cohen, 1984) with a double-step target paradigm (McLaughlin, 1967) in a dual task involving both attention and saccade shifts. To reduce as much as possible the interference with the saccade (visual) targets, we implemented a tactile version of Posner's paradigm (cue and target consisting of tactile stimuli). Indeed, we leveraged the cross-modal nature of spatial attention (Driver & Spence, 1998), notably the fact that orienting spatial attention to tactile stimuli boosts visual processing. We hypothesized that tactile spatial attention would facilitate saccadic adaptation, similar to the facilitatory effect of visual attention proposed by Gerardin et al. (2015). In this dual task, the orienting of attention toward an area of the peri-personal space was induced by a tactile cue delivered on the fingers and measured by the difference in the speeded response to a second tactile stimulation delivered on the cued side (valid trials) or the opposite side (invalid trials). The specific parameters of both the spatial attention orienting and the saccadic adaptation procedures were adapted across two experiments to investigate the effect of exogenous tactile spatial attention on the adaptation of RS (Experiment 1) and that of endogenous tactile spatial attention on the adaptation of VS (Experiment 2). Based on the hypothesis of a functional relationship between saccadic adaptation and attention, we predicted that in both cases, the orienting of tactile spatial attention would affect the adaptation of saccades.

## General methods

### Sample size estimation

We calculated our sample size with the open-access software G*Power 3.1.9.7, using an effect size based on the results obtained by Gerardin et al. (2015). In that study, saccadic adaptation was compared between two conditions (low attentional demand [LAD] vs. high attentional demand [HAD]), as assessed by the saccadic gain difference between the pre- and postexposure phases (mean ± standard deviation: LAD = −0.10 ± 0.034, HAD = −0.13 ± 0.033). Using the resulting effect size of 0.447 (other G*Power parameters: α = 0.05, power = 0.8, nonsphericity correction *Ɛ* = 1, and correlation among repeated measures = 0.5), the recommended sample size was 12 participants for this study. As our task was meant to more specifically orient spatial attention toward or away from the saccade target, we cautiously increased the number of participants to 20 per experiment.

Inclusion criteria were normal or corrected-to-normal vision; no simultaneous participation in other experiments involving sensorimotor adaptation; no history of neurological, psychiatric, or cognitive disorders preventing the comprehension of the instructions; no consumption of psychotropic drugs or alcohol; and no severe sleep deprivation during the past 24 hours. Participants were asked not to wear high heels (so they could be comfortable using a pair of response pedals) and not to use makeup or remove it before the experiment (to facilitate eye tracker setup and improve eye movement data).

### Ethical statement

The study adheres to the World Medical Association's code of ethics and the 2008 Declaration of Helsinki and received approval from the INSERM Ethics Committee (notice 21–762, IRB00003888). All participants were given 15 euros per session for their participation.

### Experimental setup

The experiment was performed in a dimly lit room. Participants were seated on a chair facing a computer screen (1,920 × 1,080 pixels, 53.5 × 31.5 cm; 144 Hz refresh rate) positioned 35 cm from their eyes and tilted backward by ∼35° from the vertical plane. Their head was stabilized using a chin rest and forehead support, and their hands were placed on a tilted support behind the screen at respective horizontal angles of about −30° and +30° relative to the body midline. Visual targets consisted of a light gray circle (0.5° of visual angle) shown on the computer screen against a dark gray background. The position of the right eye was recorded at a 1000 Hz frequency using the tower configuration of the EyeLink 1000 infrared tracker (SR Research EyeLink 1000).

Each session started with a calibration of the eye tracker by asking participants to fixate on five targets located at the center and near the screen's borders. In Experiment 1, two electrode pairs (Ambu Neuroline 700; connector: 1.5 mm; Length: 150 cm/60″) were attached to the distal phalanxes of each participant's index finger, and each pair was connected to a stimulator delivering constant current electro-cutaneous stimulations (Digitimer DS7A, controlled by the parallel port of the display computer). In Experiment 2, four pairs of the same electrodes were used (two pairs on each hand, one on the index, and one on the thumb), each electrode pair being controlled by one of four stimulators.

The supra-threshold stimulation intensity was manually adjusted to be detectable easily and comfortably. Participants were asked to steadily press two pedals (under the toes and the heel) with their right foot and to provide their answer to the tactile target stimulation by releasing one of the two pedals (connected to the parallel port of the display computer). Visual targets and stimulators were controlled by a computer program running in the Experiment Builder environment (SR Research), and signals from the eye tracker and the pedals were sampled through the same program and stored at 1000 Hz for offline analyses.

## Experiment 1: The effect of exogenous spatial attention on the adaptation of reactive saccades

### Materials and methods

#### Participants

Twenty participants were recruited for this experiment, including 11 women (one left-handed) and 9 men (all right-handed), with a mean age of 22.85 ± 3.84 years (range 18–31).

#### Design

The central part of the experiment consisted of the Exposure phase (320 trials), during which RS adaptation and exogenous spatial attention orienting were simultaneously elicited using modified versions of, respectively, the “double-step” paradigm (McLaughlin, 1967) and the exogenous attention paradigm (Posner, 1980; Posner & Cohen, 1984). The double-step paradigm consists of repeatedly inducing a postsaccadic error by displacing the visual target during saccade execution (see Introduction). In parallel, the orienting of exogenous attention was induced by a first tactile stimulus (tactile cue) delivered on one hand, and its effect was measured by asking participants to report as fast as possible with foot pedals the location of a second tactile stimulus (tactile target) delivered randomly on either hand.

As represented in Table 1, saccades directed toward the left or the right hemifield (equiprobable) were randomly elicited. Whereas half of the leftward saccades (25% in total) were exposed to an amplitude-shortening adaptation (backward double-step target procedure), the remaining leftward saccades and all the rightward (control direction) saccades were performed to a target disappearing after the saccade onset (no jump). Tactile exogenous attention was also randomly directed toward the left or the right hemifield (equiprobable). Each participant performed two sessions, during which spatial attention in the adapted leftward saccade trials was systematically directed either to the left (tactile cue on left hand, “IPSI” session) or to the right (tactile cue on right hand, “CONTRA” session). Specifically, during the IPSI session, trials with a tactile cue on the left were associated with a systematic double step of the saccadic target when presented on the left (to induce an adaptation of the saccades directed ipsilaterally to the attentional displacement) and with a disappearance of the saccadic target when shown on the right. In contrast, trials with a tactile cue on the right were associated with the disappearance of all saccadic targets (left and right). During the “CONTRA” session, the association was made between attentional cueing on the right and a systematic double step of the saccadic target presented on the left (to induce adaptation of the saccades directed contralaterally to the attentional displacement), and the saccadic target disappeared in all other trials.

**Table 1.** The different trial types in the exposure phase of Experiment 1. Trials where the visual target stepped a second time (ON) or disappeared (OFF) during the saccade are shown for the IPSI and CONTRA sessions.

Tactile cue	Left hand				Right hand			
Saccade target (T1)	Left		Right		Left		Right	
Second step (T2): IPSI session	**ON**		OFF		OFF		OFF	
Second step (T2): CONTRA session	OFF		OFF		**ON**		OFF	
Tactile target	Left hand (Valid 50%)	Right hand (Invalid 50%)	Left hand (Valid 50%)	Right hand (Invalid 50%)	Left hand (Invalid 50%)	Right hand (Valid 50%)	Left hand (Invalid 50%)	Right hand (Valid 50%)

The order of the sessions was counterbalanced between participants: 10 participants performed the IPSI session first, and 10 started with the CONTRA session. Knowing that the retention of saccadic adaptation can remain statistically significant for up to 5 days (Alahyane & Pélisson, 2005), the two sessions were separated by at least 7 days. The Exposure phase was divided into four blocks of 80 trials each, between which participants could take a short break. Feedback about the discrimination task performance (number of correct answers and mean reaction time) was presented on the computer screen at the end of each block.

In each session, the Exposure phase was preceded and followed by, respectively, a PRE-exposure phase and a POST-exposure phase, identical to each other and between the two experimental sessions (except for the random ordering of the target appearance in the right or left hemifield). Each PRE-exposure and POST-exposure phase consisted of 30 simple RSs toward a target disappearing after the saccade onset (15 rightward and 15 leftward saccades in random order).

Before starting the experiment, a short practice session was performed to accustom participants to the double task (oculomotor response and tactile discrimination) and ensure they complied with the instructions. This session was identical to the Exposure phase of the experiment but without any adaptation (the visual target disappeared at the time of saccade onset in all trials).

#### Procedure

##### Saccadic tasks

All PRE- and POST-exposure phase trials started with participants fixating on a central fixation point (FP) for 1,000 ms. As soon as a peripheral target (T1) replaced the fixation point to its left or right (eccentricity of 15° of visual angle), participants had to perform a saccade toward it as rapidly and as precisely as possible. Once the saccade onset was detected (30°/s velocity threshold), the peripheral target T1 disappeared. Participants had to maintain their gaze on the T1 location until the fixation point reappeared at the center, 200 ms following the saccade's offset. An intertrial period of 1,500 ms with the fixation point still present allowed the participant to blink if needed and to prepare for the subsequent trial.

**Figure 1.**
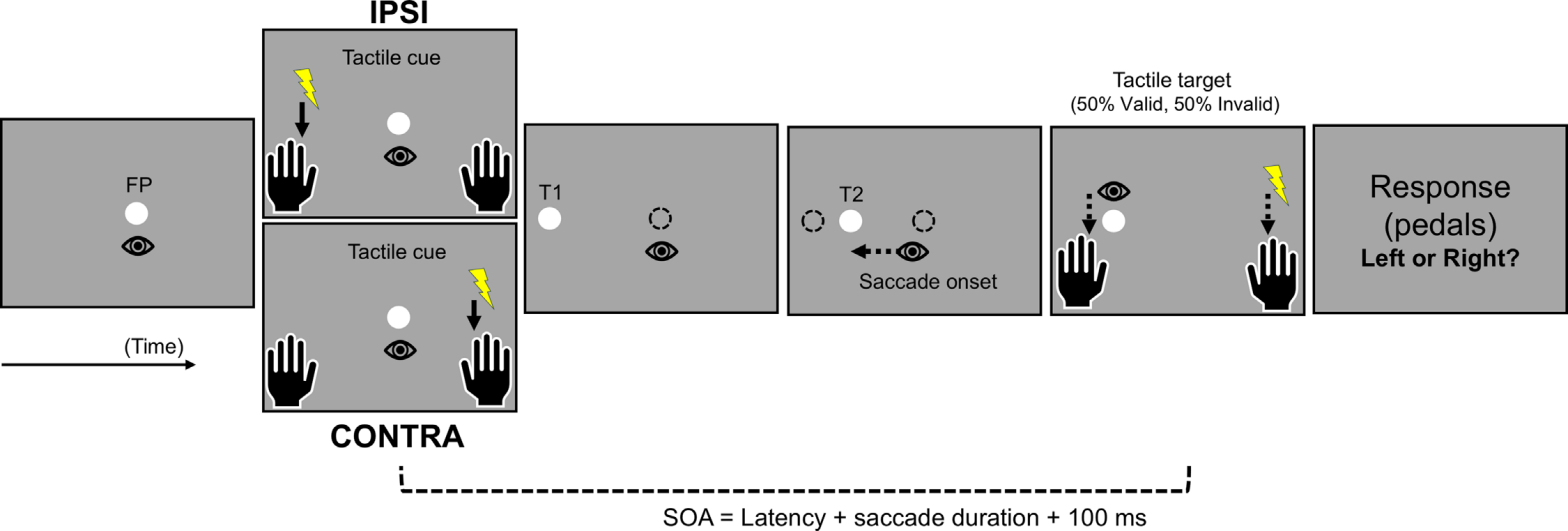
Schematic representation of the experimental protocol for leftward-adapted saccades in the exposure phase of Experiment 1. After a random fixation time, participants’ tactile exogenous attention was oriented using a tactile cue delivered on the index finger of the left hand (in IPSI session) or the right hand (in CONTRA session) 50 ms before the appearance of the saccade target T1 (15° of eccentricity). Once the onset of the saccade toward T1 was detected, T1 was replaced by a second visual target, T2 (11° of eccentricity). Then, 50 ms after the detection of saccade offset, a tactile target was delivered on the cued (Valid trials, 50%) or the uncued hand (Invalid trials, 50%). Participants had to report the location of the tactile target (left or right) using a pair of pedals placed under their right foot.

At the beginning of each trial of the Exposure phase, participants had to fixate the FP for 1,000 ms. Then, a peripheral saccade target (target T1) replaced the FP, located 15° away to the left or right. For leftward adaptation trials, the target jumped as soon as the reactive saccade was detected: T1 (15° to the left) disappeared and was replaced by T2 (11° to the left), representing a 4° jump in the direction opposite to the saccade (backward jump; see Figure 1). T2 turned off 100 ms after the saccade offset. For all other trials (no-jump), T1 disappeared at the saccade onset until the FP's reappearance.

##### Tactile discrimination task

In all trials of the Exposure phase (see Figure 1), two tactile stimulations were delivered to induce an exogenous orienting of spatial attention: (a) A tactile cue was delivered 50 ms before the appearance of the saccadic target T1 on the index finger of one hand, and (b) a tactile target was delivered, 50 ms after the saccade offset, on the index finger of the cued hand (valid trials) or the other hand (invalid trials). Participants were instructed to report the tactile target's location (which hand) as quickly as possible by releasing one of the two pedals. Ten participants released the heel pedal for the left hand and the toe pedal for the right hand, and the remaining 10 participants were assigned to the opposite combination (both groups being equally divided across the two testing orders: IPSI-first vs. CONTRA-first). We used an orthogonal stimulation–response mapping to prevent the stimulus–response spatial compatibility effect (Simon, 1990) and thus to specifically reveal attentional effects on target processing (Spence, Nicholls, Gillespie, & Driver, 1998). Once the response was detected or the 850-ms timeout had elapsed, the central fixation point appeared again for a 1,500-ms intertrial period, allowing the participant to blink and prepare for the subsequent trial.

#### Analysis

Eye position data were analyzed offline using MATLAB (MathWorks) code developed in our laboratory. This software automatically detects the beginning and end of saccades based on a velocity threshold (15*°*/s) and allows for manual corrections whenever necessary (blinks, artifacts, etc.). Saccade amplitude was measured as the difference between eye positions 50 ms before the saccade onset and 50 ms after the saccade offset. Saccade gain was calculated as the ratio between the saccade amplitude and the target initial eccentricity (distance between target T1 and saccade starting positions). The slope of the gain change during time was calculated separately for rightward and leftward saccades of the Exposure phase. Saccade's latency was calculated as the time between the appearance of T1 and the saccade onset.

Trials were excluded from the analysis when a blink occurred during the primary saccade, as well as when the saccade gain was outside the mean ± 2.5 standard deviations or when the saccade latency was less than 80 ms or outside the mean ± 3 standard deviations (means and standard deviations calculated for each participant and across each hemifield, phase, and session). This led to the exclusion of 4.73% of all trials.

Tactile discrimination performance was evaluated by the speed of correct responses (reaction time [RT]), with trials with no or incorrect responses excluded. We used the Tukey method to check whether individual participants qualified as outliers according to their mean percentages of correct responses (Chica, Sanabria, Lupiáñez, & Spence, 2007) and found that none of our participants were considered outliers (median percentage of correct answers: IPSI = 75.47%, CONTRA = 80.47%). We then calculated the difference in mean tactile discrimination RT between invalid trials and valid trials. This “tactile validity index” reflects the temporal benefit of tactile discrimination responses brought about by tactile exogenous attention.

Statistical analyses were performed using SPSS software (IBM SPSS Statistics). We first performed two analyses on saccade latency. The first test in the PRE-exposure phase examined the effect of sessions and saccade direction by submitting the median saccade latency to a two-way repeated-measures (rm) analysis of variance (ANOVA) with the Session (IPSI vs. CONTRA) and the Saccade direction (Leftward vs. Rightward) as within-subjects factors. The second looked for a potential effect of the tactile cue delivered 50 ms before the saccade target on the latency of saccades recorded in the PRE-exposure and the Exposure phases, using a three-way rm-ANOVA with the factors “Session” (IPSI vs. CONTRA), “Saccade direction” (Leftward vs. Rightward), and “Tactile cue” (Same side as saccade target vs. Opposite side relative to saccade target vs. None [Saccades in PRE-exposure phase with no cue]) as within-subjects factors.

Then, to test our main hypothesis, we evaluated the effect of attentional cueing on saccadic adaptation time course during Exposure (slope of gain change) and adaptation after-effect in the POST- relative to the PRE-exposure phases (gain) as follows. The slope of gain change during the Exposure phase was submitted to a two-way rm-ANOVA with “Session” (IPSI vs. CONTRA) and “Saccade direction” (Leftward-Adapted vs. Rightward-Control) as within-subjects factors. The saccadic gain in PRE- and POST-exposure phases was submitted to a similar rm-ANOVA with the “Phase” (PRE vs. POST) as an additional within-subjects factor (three-way rm ANOVA: Session × Saccade direction × Phase).

We also analyzed the speed of discrimination responses to check whether tactile exogenous spatial attention was successfully oriented toward the cued locations. To this aim, we submitted the RT of correct discrimination responses to the following four-way rm-ANOVA: “Session” (IPSI vs. CONTRA) × “Cue direction” (Right vs. Left) × “Saccade direction” (Rightward vs. Leftward) × “Validity” (Valid vs. Invalid).

We tested the effect of session and pedal counterbalancing across participants on the saccadic gain and the discrimination RT, respectively, by including them as additional between-subjects factors in the above analyses.

We calculated the ratio of gain change in each session and each saccade direction as follows: Gain change ratio = (Gain PRE – Gain POST) / (Gain PRE) (e.g., a gain change ratio of +0.2 corresponds to a 20% decrease in saccadic gain in the POST-exposure compared to the PRE-exposure phase). We then separately correlated (one-sided Pearson's correlation), for the leftward-adapted trials, the tactile validity index with the gain change ratio and the gain change slope to investigate whether the strength of adaptation depended on the efficiency of attentional shifts, as measured by participants’ discrimination performance.

When the sphericity was violated in the ANOVAs, Greenhouse–Geisser correction was applied. Results were considered significant when *p* < 0.05 at *α* = 0.05.

### Results

#### Saccade latency in PRE-exposure

The latency of saccades in the PRE-exposure phase did not differ between the two sessions or between the two saccade directions, as disclosed by the Session × Saccade direction rm-ANOVA (no significant main effect or interaction, all *F* ≤ 2.551, all *p*s ≥ 0.127). The grand mean of the median saccade latencies was 185.01 ± 18.64 ms, which is consistent with the latency of RS (Becker, 1989).

#### Cue effect on saccade latency

The three-way Session × Saccade direction × Tactile cue rm-ANOVA showed only a significant main effect of the Tactile cue factor (*F*(1.226, 23.303) = 16.751, *p* < 0.001; all others, *F* ≤ 3.761, *p*s ≥ 0.067). Pairwise comparisons showed that the saccade median latency was 6 ms lower when the cue was ipsilateral versus contralateral to the saccade target (*p* = 0.002) and was in both cases lower than with no cue (PRE-exposure phase), respectively, by 18 ms for ipsilateral cues (*p* < 0.001) and by 12 ms for contralateral cues (*p* = 0.018). Crucially, this acceleration of saccade onset by the tactile cue did not interact with Session or Saccade direction.

#### The slope of gain change

**Figure 2.**
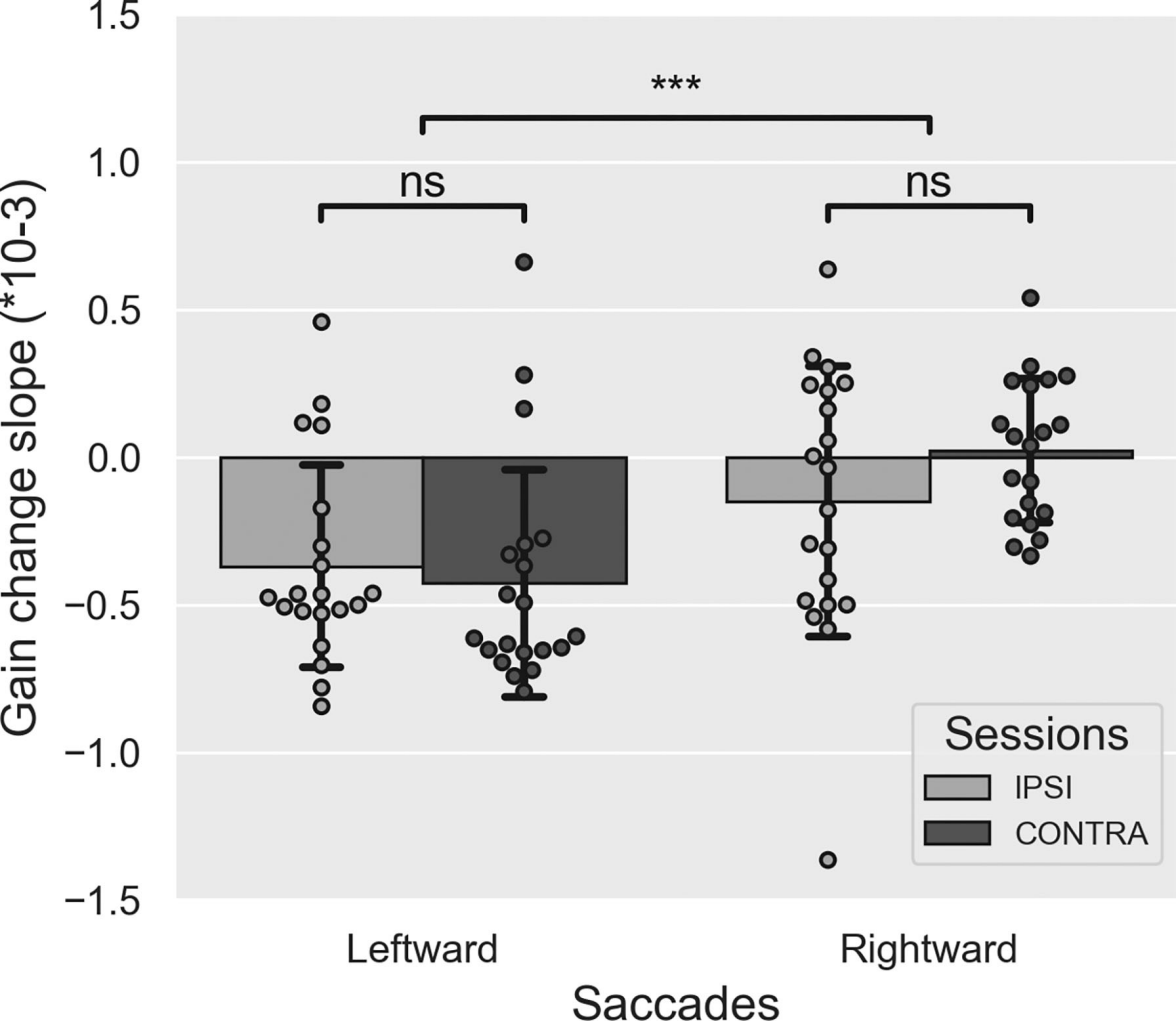
Saccade gain change during Exposure in Experiment 1. Mean slope (± *SD*) of the linear relationship between saccadic gain and trial number in the Exposure phase, separately for leftward and rightward saccades and for IPSI (light gray) and CONTRA sessions (dark gray). *t*-tests: ^ns^*p* > 0.05, ***p < 0.001.

The mean slope values of gain change measured during Exposure are plotted in Figure 2. The two-way Session × Saccade direction rm-ANOVA showed a significant main effect of Saccade direction (*F*(1, 19) = 14.918, *p* = 0.001; mean slope ± *SD*: Leftward-Adapted saccades = −0.3987 × 10^−3^ ± 0.345 × 10^−3^, Rightward-Control saccades = −0.064 × 10^−3^ ± 0.278 × 10^−3^). There was neither a significant main effect of Session (*F*(1, 19) = 0.935, *p* = 0.346) nor a significant Session × Saccade direction interaction (*F*(1, 19) = 3.749, *p* = 0.068). These results show that the gain of leftward RS decreased during exposure due to the repetition of backward double-step trials and, in comparison, that the gain of rightward RS toward no-jump targets remained stable.

To test if there was an effect of the session order on the gain slopes, we performed a separate rm-ANOVA similar to the previous one but with an additional between-subjects factor, the “Group” (IPSI-first vs. CONTRA-first) factor. This analysis showed no significant main effect or interaction of this “Group” factor (all *F* ≤ 4.076, all *p*s ≥ 0.059). Altogether, these findings are consistent with the adaptation of leftward RS, which, however, did not differ between the IPSI and CONTRA sessions.

#### Gain variation

**Figure 3.**
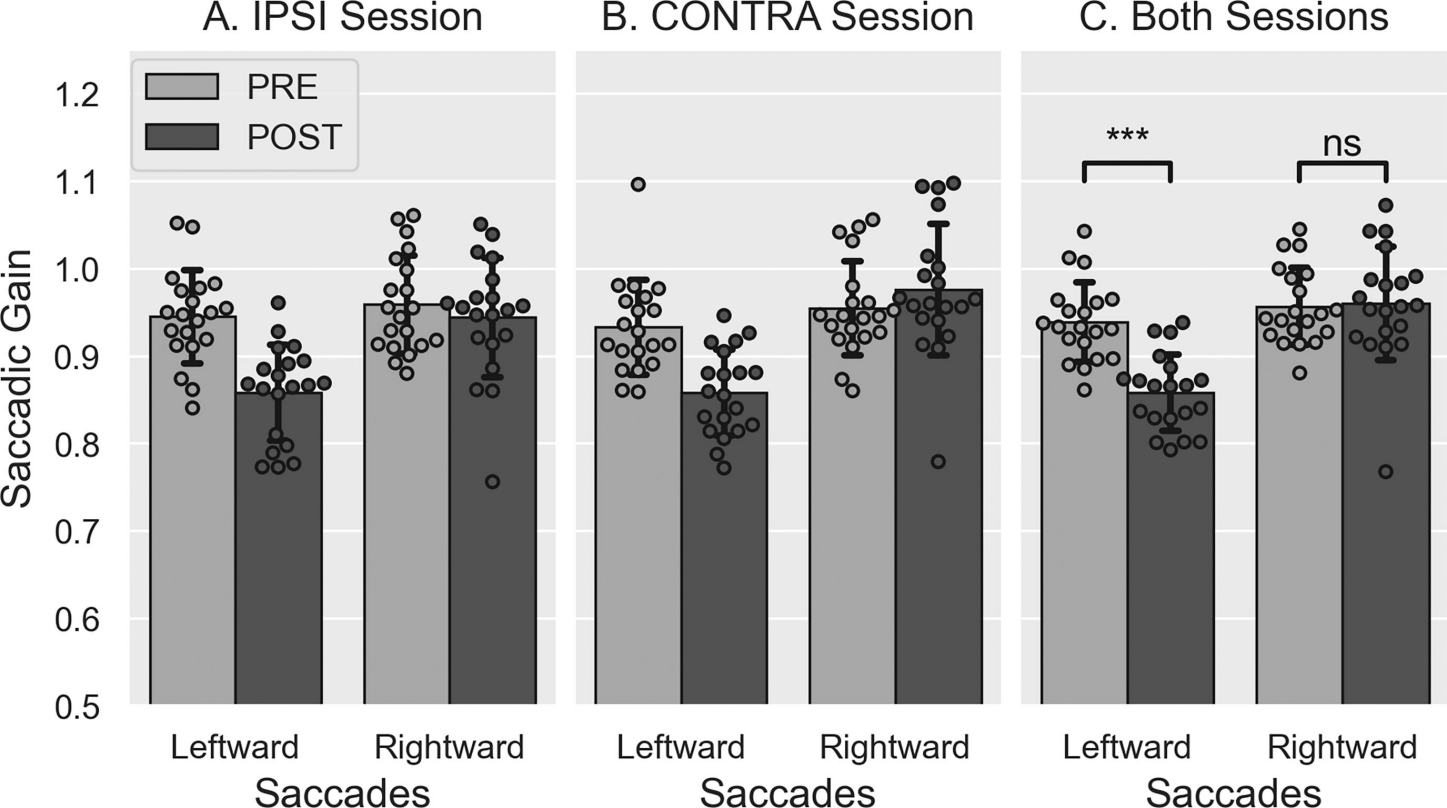
Saccade gain in PRE- and POST-exposure in Experiment 1. Mean saccadic gain (± *SD*) of leftward and rightward saccades in the PRE (light gray) and POST phases (dark gray), in IPSI (**A**) and CONTRA sessions (**B**) and both sessions pooled together (**C**). *t*-tests: ^ns^*p* > 0.05, ***p < 0.001.

The mean gain values measured in the PRE- and POST-exposure phases are illustrated in Figure 3. The three-way Session × Saccade direction × Phase rm-ANOVA revealed a significant main effect of the factors Phase (*F*(1, 19) = 27.636, *p* < 0.001) and Saccade direction (*F*(1, 19) = 23.150, *p* < 0.001), as well as significant Session × Phase (*F*(1, 19) = 10.563, *p* = 0.004) and Phase × Saccade direction interactions (*F*(1, 19) = 51.061, *p* < 0.001). No other significant effect or interactions were found (all *F* ≤ 2.113, all *p*s ≥ 0.162). Post hoc pairwise Bonferroni comparisons assessing the Session × Phase interaction showed no significant difference of gain between sessions in the PRE-exposure (*p* = 0.452) and the POST-exposure phases (*p* = 0.166), while revealing significant differences between PRE- and POST-exposure phases in both the IPSI (*p* < 0.001) and the CONTRA sessions (*p* = 0.002). Post hoc pairwise Bonferroni comparisons investigating the significant Phase × Saccade direction interaction revealed significant differences between phases for leftward-adapted saccades (mean gain ± *SD*: PRE: 0.939 ± 0.045, POST: 0.858 ± 0.044, *p* < 0.001) but not for rightward-control saccades (PRE: 0.956 ± 0.044, POST: 0.960 ± 0.065, *p* = 0.731), also showing significant differences between Saccade direction in the POST-exposure (*p* < 0.001) but not the PRE-exposure (*p* = 0.196) phases.

Next, we verified whether the session order affected the above results by submitting the saccade gain to the same ANOVA with the additional between-subjects factor “Group” (IPSI-first vs. CONTRA-first). This four-way ANOVA showed no significant main effect of this Group factor (*F*(1, 18) = 0.160, *p* = 0.694) but a significant Group × Saccade direction interaction (*F*(1, 18) = 6.285, *p* = 0.022). Post hoc pairwise Bonferroni comparisons showed a significant gain difference between the groups for leftward (*p* = 0.045) but not rightward saccades (*p* = 0.379), as well as a significant gain difference between saccade directions in the IPSI-first group (*p* < 0.001) but not in the CONTRA-first group (*p* = 0.053). None of the interactions between the group factor and the other within-subjects factors were significant (all *F* ≤ 2.594, all *p*s ≥ 0.125).

These results are consistent with and confirm those reported above for the gain slope, indicating that, as expected, backward adaptation after-effects were successfully induced for leftward (double-step trials) but not rightward RS (no jump trials). However, the absence of triple-interaction Session × Phase × Saccade direction does not support our hypothesis of a stronger adaptive after-effect of leftward RS in the IPSI versus CONTRA sessions.

#### Discrimination RT

**Figure 4.**
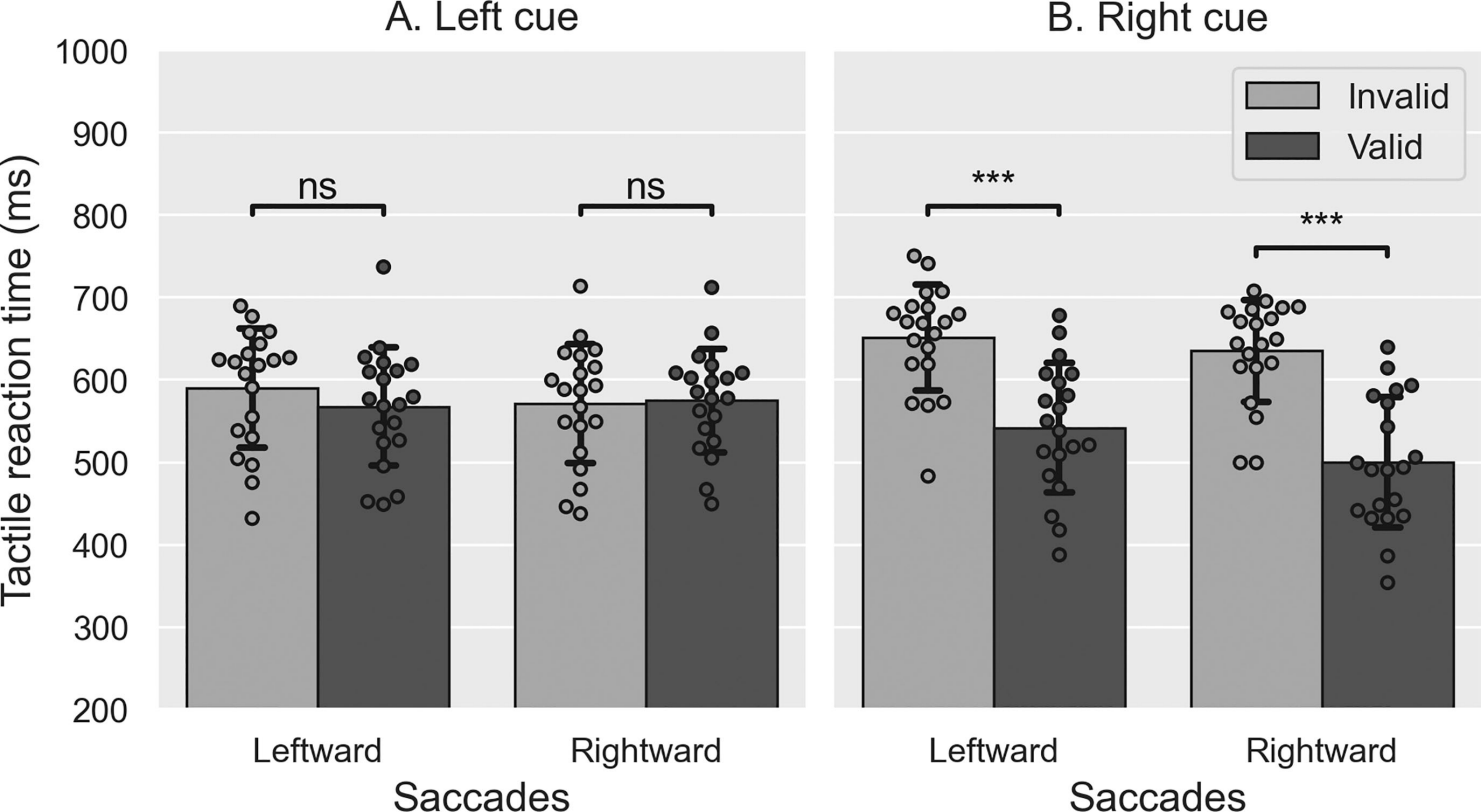
Tactile discrimination performance in Experiment 1. Mean reaction time to the tactile target (± *SD*) in leftward and rightward saccade trials for valid (dark gray) and invalid trials (light gray) when the tactile cue was delivered on the left (**A**) or the right (**B**) hand. *t*-tests: ^ns^*p* > 0.05, ***p < 0.001.

To ensure that participants’ spatial attention was successfully oriented toward the tactile cue in both the IPSI and CONTRA sessions, we analyzed the RT of their correct discrimination responses to the tactile targets. The mean RT values measured in the Exposure phase are plotted in Figure 4. The four-way rm-ANOVA (Session × Validity × Cue direction × Saccade direction) showed significant main effects of the factors Saccade direction (*F*(1, 19) = 16.030, *p* < 0.001, corresponding to a 17.6-ms faster discrimination of the tactile target location in rightward saccade trials than in leftward saccade trials) and Validity (*F*(1, 19) = 139.310, *p* < 0.001, with a 65.9-ms faster discrimination in valid trials than in invalid trials). The two-way interactions involving the Cue direction factor were all significant: Cue direction × Session (*F*(1, 19) = 4.970, *p* = 0.038), Cue direction × Saccade direction (*F*(1, 19) = 19.373, *p* < 0.001), and Cue direction × Validity (*F*(1, 19) = 24.816, *p* < 0.001). The following three-way interactions were also significant: Session × Cue direction × Saccade direction (*F*(1, 19) = 13.842, *p* = 0.001) and Cue direction × Saccade direction × Validity (*F*(1, 19) = 7.358, *p* = 0.014). The ANOVA showed no other significant main effects or interactions (all *F* ≤ 1.694, all *p*s ≥ 0.209). Post hoc pairwise Bonferroni comparisons assessing the Session × Cue direction × Saccade direction interaction showed no significant difference in RT between sessions for saccades or cue directions. In addition, they showed that for leftward saccades in the CONTRA session, participants responded significantly faster when the cue was delivered on the left hand than when the cue was delivered on the right hand (36-ms difference, *p* < 0.001). The cue direction had no further significant effect (all *p*s ≥ 0.168). Finally, this post hoc analysis showed that participants were significantly faster in discriminating the target location after rightward saccades (all *p*s ≤ 0.018) except in the CONTRA session, when the cue was presented on the left hand (*p* = 0.665). A second series of post hoc pairwise Bonferroni comparisons was performed to investigate the Cue direction × Saccade direction × Validity interaction. It revealed that participants were significantly faster at discriminating the target location after rightward versus leftward saccades (all *p*s ≤ 0.034), except for valid trials when the cue was delivered on the left hand (*p* = 0.314). In addition, there was a significant validity effect with right cues for both saccades directions (all *p*s ≤ 0.001; Figure 4B) but not with left cues (all *p*s ≥ 0.115; Figure 4A), with participants being faster in discriminating the target location in valid trials than invalid ones, but only when the cue was delivered on the right hand. In summary, participants’ tactile exogenous attention was successfully oriented when the cue was presented to the right but not to the left, an asymmetry observed in both IPSI and CONTRA sessions. Thus, these discrimination RT results confirm that tactile exogenous spatial attention was successfully oriented away from the (leftward)-adapted saccades in the CONTRA session, but they provide no evidence that it was oriented toward the adapted saccades in the IPSI session.

We also checked whether pedal assignment had an effect on the discrimination RT results. To do so, we performed a new ANOVA similar to the above analysis, except here we included a between-subjects factor, “Toes pedal” (Left vs. Right), representing which tactile target the participants designated by releasing the pedal under the toes. Results showed no significant main effect of the “Toes pedal” factor (*F*(1, 18) = 0.425, *p* = 0.523), but this factor significantly interacted with the “Saccade direction” (*F*(1, 18) = 13.364, *p* = 0.002; when the pedal under the toes was assigned to left tactile targets, participants responded 20 ms faster after performing leftward saccades compared to rightward saccades), as well as with the “Cue direction” (*F*(1, 18) = 4.944, *p* = 0.039; when the pedal under the toes was assigned to right tactile targets, participants responded 26 ms faster after a rightward cue in comparison to a leftward one). There were no other significant interactions involving the between-subjects factor “Toes pedal” (all *F* ≤ 3.619, all *p*s ≥ 0.073).

#### Correlations

**Figure 5.**
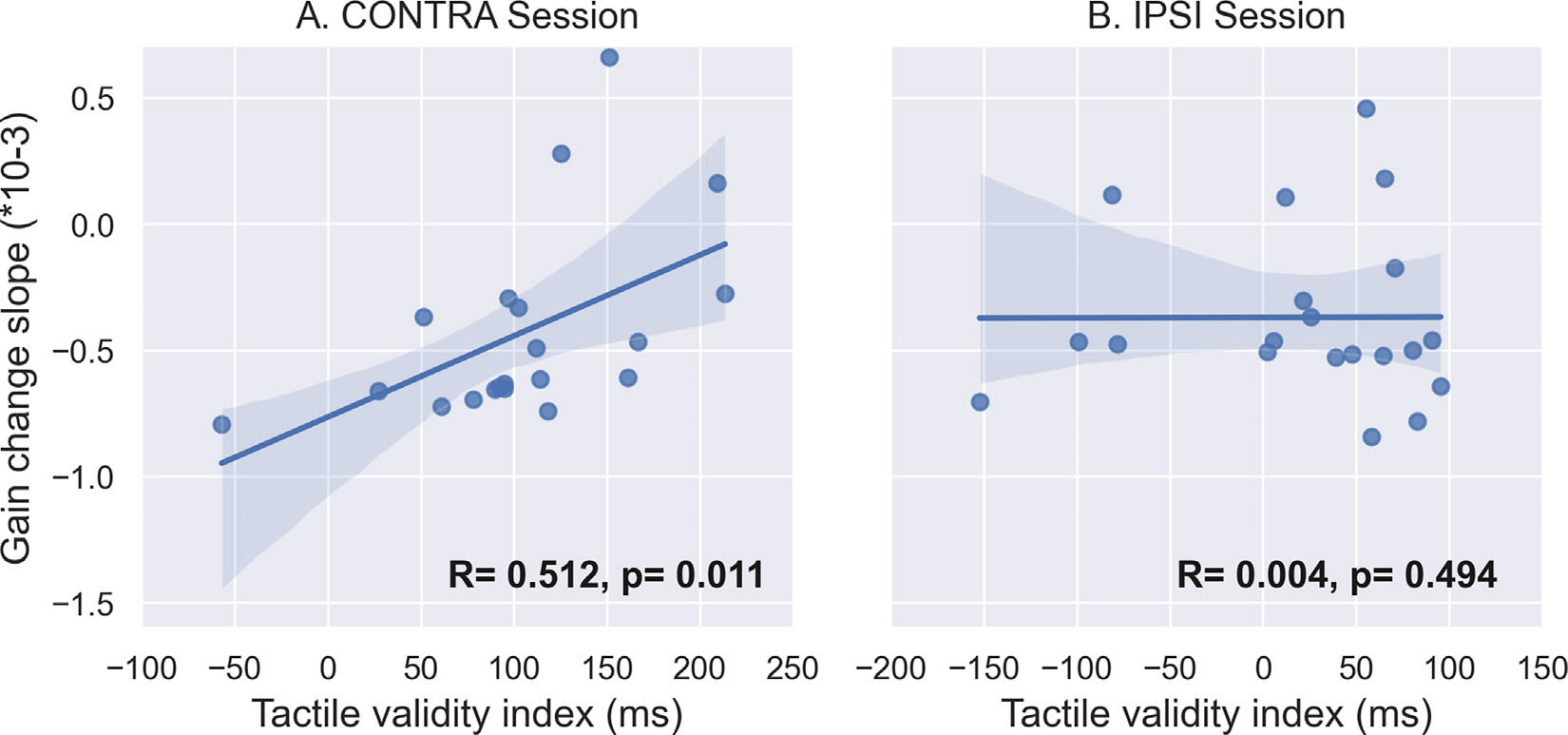
Correlation between tactile discrimination performance and saccadic gain change slope in Experiment 1. Correlation between the tactile validity index (ms) and the slope of gain change during Exposure for leftward saccade trials in the CONTRA (**A**) and IPSI (**B**) sessions. *R* represents Pearson's correlation value, and *p* represents the *p*-value of the one-sided Pearson correlation.

**Figure 6.**
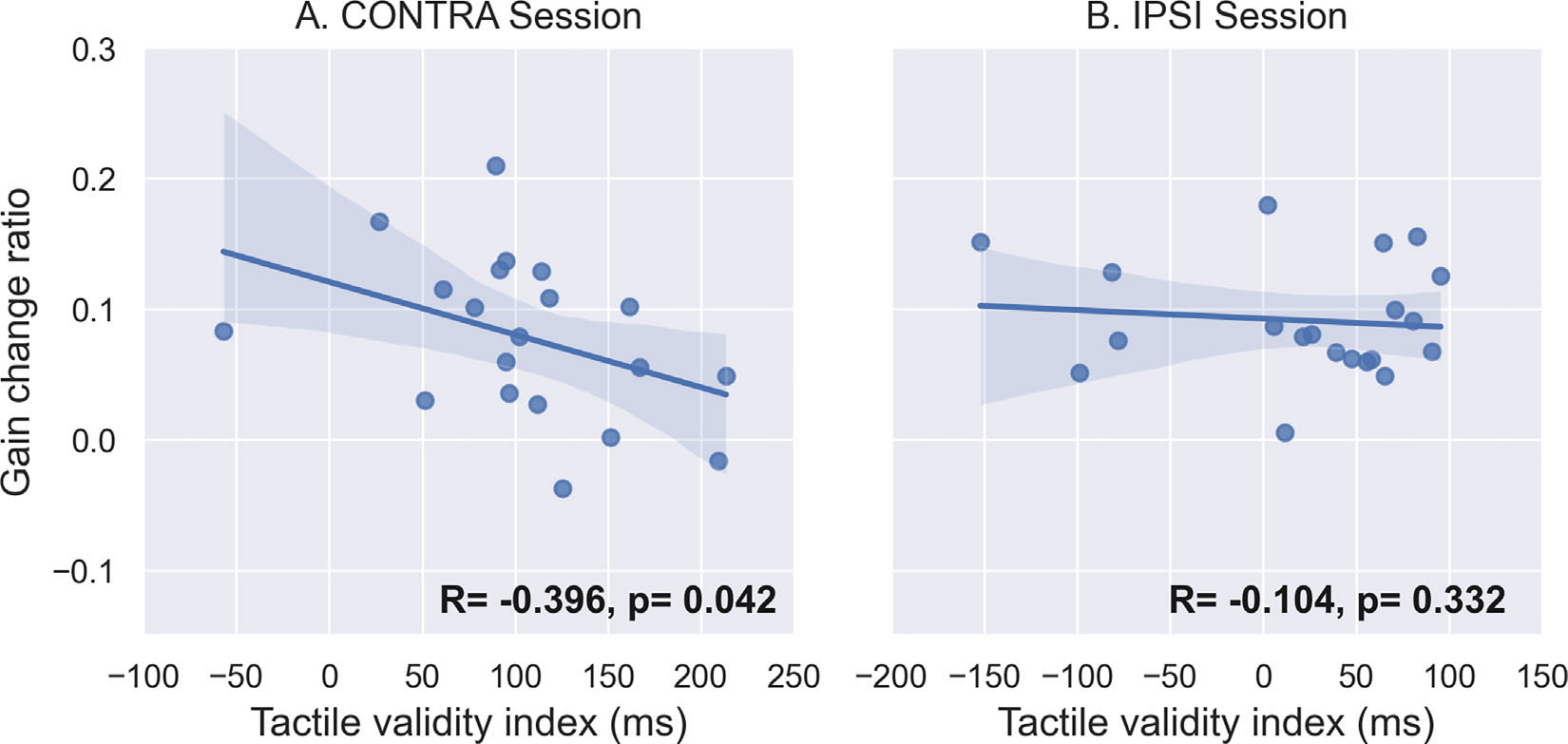
Correlation between tactile discrimination performance and saccadic gain change ratio in Experiment 1. Correlation between the tactile validity index (ms) and the gain change ratio between PRE- and POST-exposure for leftward saccades in both CONTRA (**A**) and IPSI (**B**) sessions. *R* represents Pearson's correlation value, and *p* represents the *p*-value of the one-sided Pearson correlation.

The discrimination performance described in the previous section suggests that tactile spatial attention may not have been similarly oriented in the IPSI and CONTRA sessions during the adaptive exposure of leftward saccades. Further, this group-level analysis of discrimination performance may hide some intersubject variability in the capability of shifting spatial attention. Our hypothesis of coupling between attention and adaptation would predict that participants who shifted attention more successfully should show stronger effects on the adaptation of RS. Thus, we analyzed the correlation between the tactile validity index (indexing spatial attention orienting) and the two measures of leftward saccade adaptation: gain change slope (the more negative this value is, the faster the backward adaptation is achieved) and gain change ratio (the more positive this value is, the higher the amount of adaptation achieved). These correlation analyses are illustrated in Figures 5 and 6 and further summarized in Figure 13. Starting with the CONTRA session, we found a significant positive correlation between the slope of gain change and the tactile validity index (Figure 5A, *R* = 0.512, *p* = 0.011), as well as a significant negative correlation between the gain change ratio and the tactile validity index (Figure 6A, *R* = −0.396, *p* = 0.042). In the IPSI session, the correlation analysis showed neither a significant correlation between the slope of gain change and the tactile validity index (Figure 5B, *R* = 0.004, *p* = 0.494), nor a significant correlation between the gain change ratio and the tactile validity index (Figure 6B, *R* = −0.104, *p* = 0.332).

### Interim discussion

In this experiment, we investigated the effect of exogenous orienting of spatial attention on the adaptation of leftward RS. We were able to reliably induce adaptation of those saccades (saccadic gain decreased during Exposure and remained lower in the POST- vs. PRE-exposure phases), but group-level statistical comparisons between the IPSI and CONTRA sessions did not support an effect of the direction of exogenous spatial attention on RS adaptation. However, this analysis may have been insufficiently sensitive, all the more so since participants’ tactile discrimination performance failed to reveal any successful orienting of tactile exogenous attention for leftward cues. Nevertheless, if our hypothesis is valid, the gain decrease due to saccadic adaptation should correlate with the amount of attention shifts obtained in each session. Indeed, when exogenous orienting of spatial attention was oriented successfully, namely for leftward saccades in the CONTRA session (i.e., away from the saccade's target location), the adaptation was slower and weaker (significant positive correlation with slope and negative correlation with gain change, respectively), thus providing partial support for our hypothesis. However, the second prediction of this hypothesis (IPSI session) was not supported by our results, as the absence of significant correlations for leftward saccade parameters does not argue for a facilitatory effect of exogenous attention when oriented toward the saccade's target on RS adaptation.

In Experiment 2, using the same strategy as in Experiment 1, we investigated whether endogenous shifts of spatial attention toward tactile cues could modulate the adaptation of VSs.

## Experiment 2: The effect of endogenous spatial attention on the adaptation of voluntary saccades

### Material and methods

#### Participants

Twenty new participants were recruited for this experiment, including 10 women (one left-handed) and 10 men (one left-handed), with a mean age of 26.1 ± 4.49 years (range 20–37).

#### Design

Each participant performed two sessions, with a minimum 7-day interval in between, where tactile endogenous attention in the adapted saccade trials was directed either toward (IPSI session) or away from (CONTRA session) the location of the VS target. Contrary to Experiment 1, both leftward and rightward VSs were simultaneously exposed to the adaptive double-step procedure (there was no exposure trial with the target disappearing at saccade onset); thus, the IPSI and CONTRA association between adapted saccade direction and attention orienting applied simultaneously to both hemifields (see Table 2).

**Table 2.** The different trial types in the exposure phase of Experiment 2. Saccadic adaptation was elicited by the visual target second step (ON) in both hemifields simultaneously, but with opposite spatial assignments to tactile cueing in the IPSI vs. CONTRA sessions.

Session	IPSI				CONTRA			
Tactile cue	Left hand		Right hand		Left hand		Right hand	
Saccade target (T1)	Left		Right		Right		Left	
Second step (T2)	**ON**		**ON**		**ON**		**ON**	
Tactile target	Left hand (Valid 80%)	Right hand (Invalid 20%)	Left hand (Invalid 20%)	Right hand (Valid 80%)	Left hand (Valid 80%)	Right hand (Invalid 20%)	Left hand (Invalid 20%)	Right hand (Valid 80%)

Each session comprised three phases: the PRE- and POST-exposure phases (30 trials each), consisting of simple VS toward transient visual targets (disappearing at saccade onset), and the Exposure phase (160 trials), wherein each trial combined adaptation (McLaughlin, 1967) and endogenous spatial attention-orienting paradigms (Chica et al., 2007). In the Exposure phase, the second (intrasaccadic) target step was directed backward relative to the saccade direction to induce a saccade-shortening adaptation. During all three phases, saccades directed toward the left or right hemifield were randomly elicited with equal probabilities (50% each).

The order of the IPSI and CONTRA sessions was counterbalanced among participants, with 10 starting with the former and the remaining 10 with the latter.

#### Procedure

##### Saccadic tasks

All trials of the PRE- and POST-exposure phases started with a central FP appearing for 1,200 ms. Then, a visual target T1 was presented at 15° to the left or right (FP still visible). Participants were asked to maintain fixation on FP for a random duration (average 1,100 ms), after which a green circle (diameter = 0.25°) appeared inside the fixation point, prompting participants to perform a saccade toward T1 (if a saccade was detected before this go-signal, the trial was aborted and replaced by a new one). Once the saccade onset was detected (velocity threshold = 30*°*/s), both T1 and FP disappeared, and participants were instructed to keep fixating on the location of T1 until the reappearance of FP, indicating the end of the trial.

**Figure 7.**
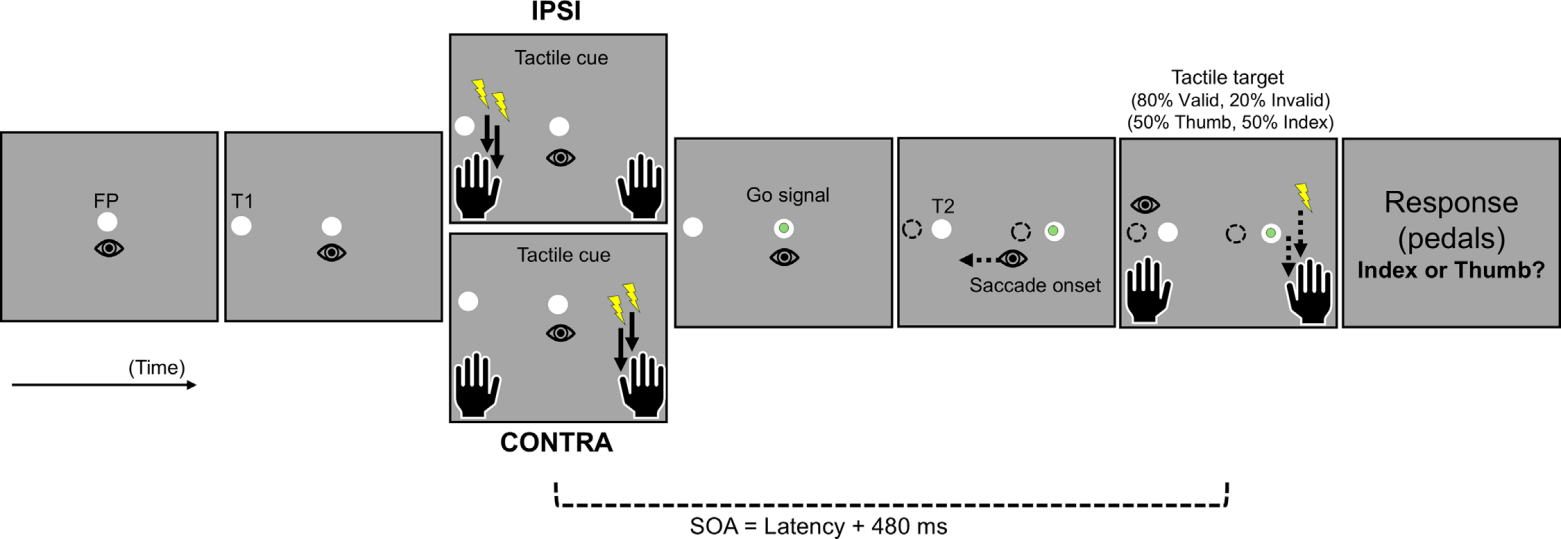
Schematic representation of the experimental protocol for leftward saccades in the exposure phase of Experiment 2. After a random fixation time (on FP), the saccade target T1 was presented at 15° eccentricity. However, the participants were instructed to maintain fixation while their tactile endogenous attention was oriented using a tactile cue delivered on both the index finger and the thumb of the left hand (in IPSI session) or the right hand (in CONTRA session); this tactile cue was provided at a random duration after T1 appearance and 380 ms before the appearance of the saccade's go-signal (green circle inside the FP). Once the onset of the saccade toward T1 was detected, the visual scene shifted 4° toward the right, thus forming a backward step. Then, 100 ms after saccade onset, a tactile target was delivered on the cued (Valid trials, 80%) or the uncued hand (Invalid trials, 20%), either on the thumb or on the index finger (50% chance each). Participants had to report the location of the tactile target (Index finger or Thumb) using a pair of pedals placed under their right foot.

In the Exposure phase, the sequence of visual events was the same as in the PRE- and POST-exposure phases until saccade detection. At that time, the saccade target T1, the fixation point FP, including the embedded go-signal, all stepped 4° in a backward direction, and participants had to maintain fixation over the stepped peripheral target (T2 located at 11°), as represented in Figure 7.

##### Tactile discrimination task

In the Exposure phase, a tactile stimulation (cue) was delivered to both the index finger and thumb of one hand at a constant interval of 380 ms preceding the go signal (i.e., at a random delay, averaging 720 ms, after T1 onset). One hundred milliseconds after the saccade onset, a second tactile stimulation (target) occurred on a single finger (index finger or thumb, 50% probability) of either the cued hand (Valid condition, 80% of the total number of trials) or the opposite, uncued hand (Invalid condition, 20% of the trials; see Figure 7). Participants used foot pedals to report as fast and accurately as possible whether the tactile target was on the index finger or thumb finger. This differs from the task in Experiment 1 (discrimination of which hand received the tactile target), in order to have a more efficient discrimination task better suited for inducing endogenous shifts of attention. Upon response (or after the 1,500-ms time-out), T2 disappeared, and performance feedback was displayed for 200 ms around the location of T2 to indicate whether their answer was correct (“+1”), “incorrect,” or “too late.” Then, a blank screen followed for 750 ms until the beginning of the subsequent trial, a period during which participants could blink if necessary. The Exposure phase was divided into two blocks of 80 trials each, followed by a short break of a few seconds, allowing the participants to rest while their mean performance (percentage of correct answers and mean RT) during the last block was displayed on the screen.

Before starting the experiment, a short practice session was performed to accustom participants to the double task (oculomotor response and tactile detection) and ensure they complied with the instructions. This session was identical to the exposure phase of the experiment but was shorter and without any adaptation elicited (T1 and FP disappeared at the saccade onset).

#### Analysis

The data processing and parameter extraction of the saccadic and tactile discrimination responses were similar to those of Experiment 1. For the saccade latency, the minimum latency of VS was set at 120 ms (instead of 80 ms for RS in Experiment 1). Overall, 10.8% of trials were excluded. The Tukey method was again used to identify participants as outliers according to their mean percentage of correct answers in the attentional task (group median: IPSI = 75.31%, CONTRA = 75.94%; Chica et al., 2007). Two participants were identified as outliers and thus removed. Therefore, the analyses described in the following were based on a final dataset of 18 participants.

We first performed two analyses on saccade latency. The first one tested in the PRE-exposure phase, using a two-way rm-ANOVA, the effect on median saccade latency of the Session (IPSI vs. CONTRA) and the Saccade direction (Leftward vs. Rightward) as within-subjects factors. The second investigated the effect of the tactile cue on saccade latency: We submitted the median latency of saccades collected in the Exposure phase, along with those measured in the PRE-exposure phase, where no tactile cues were delivered (the IPSI and CONTRA sessions were collapsed after checking the absence of a significant difference), to a two-way rm-ANOVA with the factors “Saccade direction” (Leftward vs. Rightward) and “Tactile cue” (Same side vs. Opposite side vs. None) as within-subjects factors.

Then, to test our main working hypothesis of a modulation of VS adaptation by shifts of endogenous attention, the slope of gain change during Exposure was subjected to a two-way rm-ANOVA, with Session (IPSI vs. CONTRA) and Saccade direction (Leftward vs. Rightward) as within-subjects factors. In the second ANOVA testing our hypothesis, we submitted the saccade gain measured in the PRE- and POST-exposure phases to a three-way rm-ANOVA with the factors Session (IPSI vs. CONTRA) × Saccade direction (Leftward vs. Rightward) × Phase (PRE vs. POST).

Additionally, to check whether endogenous attention was adequately oriented to the cued location, we submitted the tactile discrimination RT to a three-way rm-ANOVA with Session (IPSI vs. CONTRA), Saccade direction (Rightward vs. Leftward), and Validity (Valid vs. Invalid) as within-subjects factors.

We tested the effect of session and pedal counterbalancing across participants on the saccadic gain and the discrimination RT, respectively, by including them separately in the above analyses as additional between-subjects factors (“Group”: IPSI-first vs. CONTRA-first or “Toes pedal”: Index finger vs. Thumb).

Lastly, as in Experiment 1, we explored the correlations (one-sided Pearson's correlation) between the tactile validity index and the gain change ratio, as well as between the tactile validity index and the gain change slope. The *p*-values were adjusted for multiple testing using the false discovery rate method.

When the sphericity was violated in the ANOVAs, Greenhouse–Geisser correction was applied. Results were considered significant when *p* < 0.05 at *α* = 0.05.

### Results

#### Saccade latency in PRE-exposure

The two-way rm-ANOVA applied to the median values of saccade latency showed that the main effects of the Session and Saccade direction factors and their interaction were not significant (all *F* ≤ 0.588, all *p*s ≥ 0.454). The grand mean of saccade median latency pooled across the two saccade directions and the two sessions was equal to 274.52 ± 71.01 ms, which is consistent with the latency of VS (Nikolov, 2020).

#### Cue effect on saccade latency

The two-way Saccade direction × Tactile cue rm-ANOVA showed a significant main effect only of the Tactile cue (*F*(2, 34) = 6.272, *p* = 0.005; all other effects *F* ≤ 0.334, *p*s ≥ 0.718). Pairwise comparisons showed no significant difference in the median latency of saccades directed ipsilaterally versus contralaterally to the tactile cue but also showed that, in both cases, latency was higher than for saccades in the PRE-exposure phase with no preceding cue by, respectively, 53 ms (*p* = 0.013) and 57 ms (*p* = 0.028). Note that this increased latency of VS by tactile cues did not interact with Session or Saccade direction.

#### The slope of gain change

**Figure 8.**
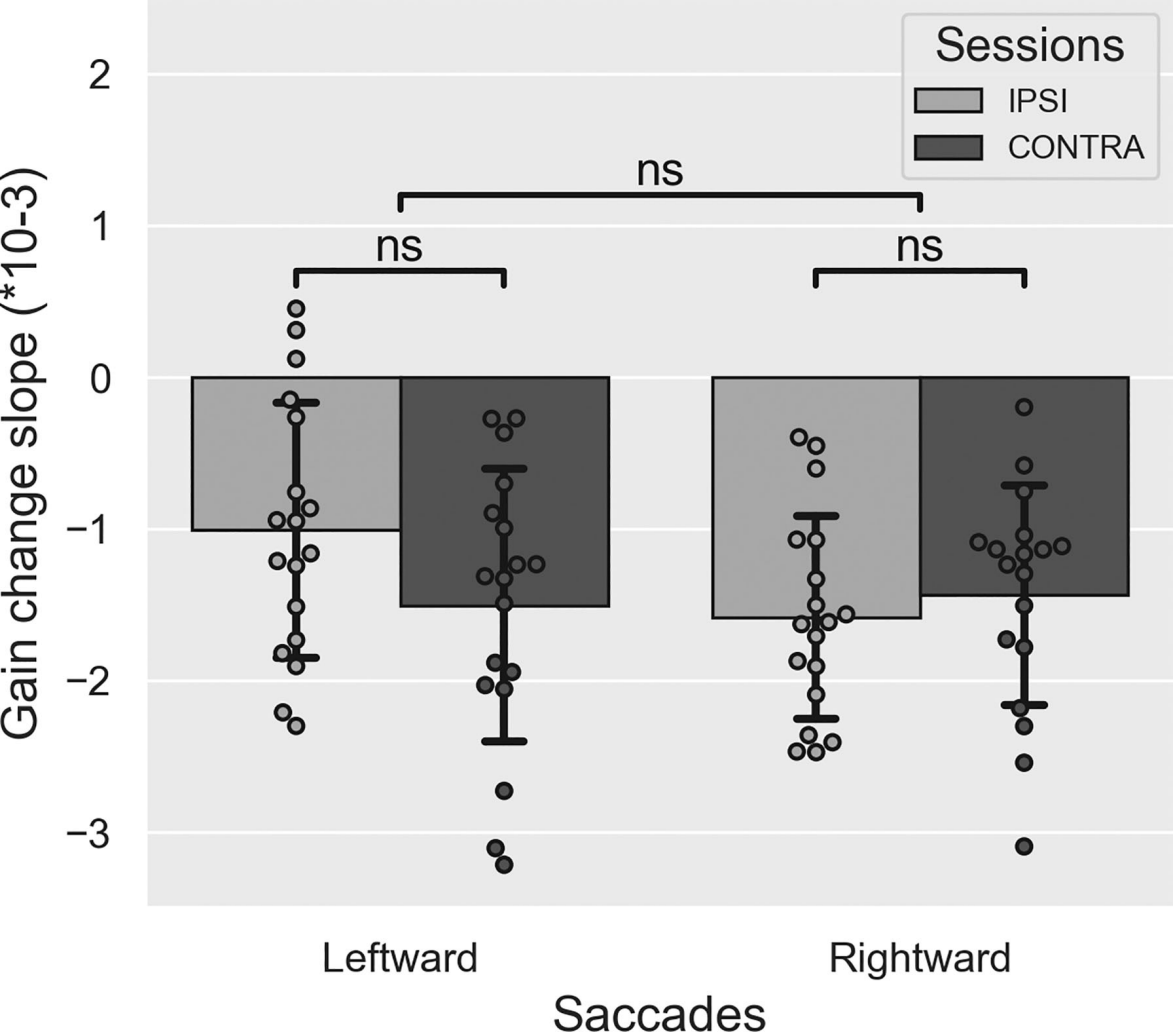
Saccade gain change during Exposure in Experiment 2. Mean slope (± *SD*) of the linear relationship between saccadic gain and trial number in the exposure phase, separately for leftward and rightward saccades and for IPSI (light gray) and CONTRA sessions (dark gray). *t*-tests: ^ns^*p* > 0.05.

The slope of gain change during Exposure is illustrated in Figure 8, showing a decreased gain for both saccade directions. This is consistent with the use of backward double-step targets for both saccade directions in this experiment. This pattern is confirmed by the two-way rm-ANOVA showing no significant main effect of Saccade direction (*F*(1, 17) = 2.076, *p* = 0.168; mean slope ± *SD*: Leftward saccades = −1.258 × 10^−3^ ± 0.658 × 10^−3^, Rightward saccades = −1.513 × 10^−3^ ± 0.329 × 10^−3^) or Session (*F*(1, 17) = 1.105, *p* = 0.308) or any significant Saccade direction × Session interaction (*F*(1, 17) = 2.052, *p* = 0.170). Comparing the mean slope to zero (one-sample *t*-test with Bonferroni correction to multiple comparisons) showed significantly negative values in both sessions and for both saccade directions (all *p*s ≤ 0.001). In addition, the mean slopes did not differ between sessions (paired *t*-tests, Leftward saccades: *p* = 0.082 and Rightward saccades: *p* = 0.618). These results show that backward adaptation was successfully induced in both sessions and for both saccade directions. However, they also did not reveal any significant difference in gain slope values between the IPSI and the CONTRA sessions.

As in Experiment 1, we tested if the session order had an effect on the slope analysis. To this aim, we performed a rm-ANOVA similar to the previous one, but with the “Group” factor (IPSI-first vs. CONTRA-first) added as a between-subjects factor. This analysis showed no significant main effect or interaction for the “Group” factor (all *F* ≤ 3.206, all *p*s ≥ 0.092).

#### Gain variation

**Figure 9.**
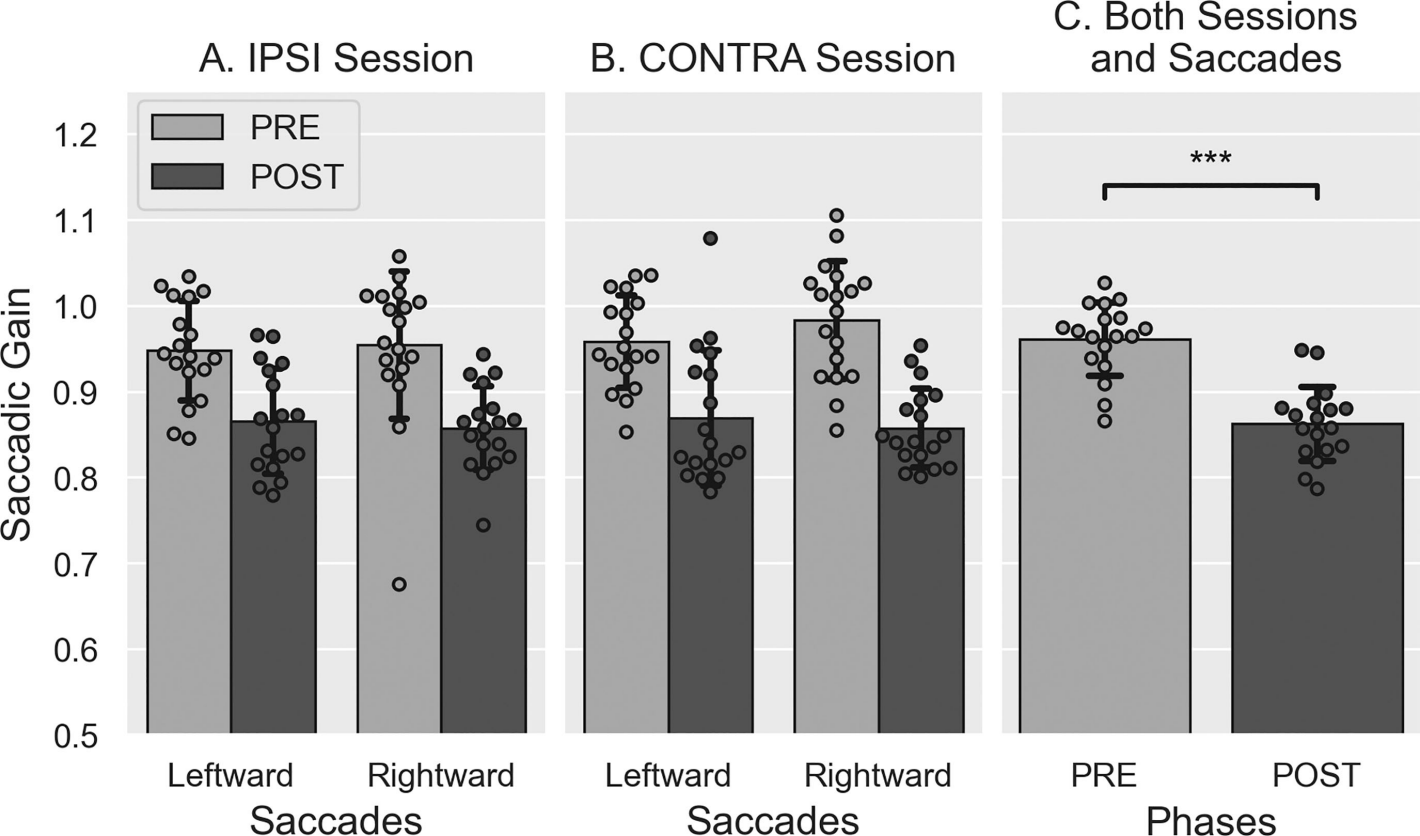
Saccade gain in PRE- and POST-exposure in Experiment 2. Mean saccadic gain (± *SD*) of leftward and rightward saccades in the PRE (light gray) and POST phases (dark gray), in IPSI (**A**) and CONTRA sessions (**B**), as well as the average PRE- and POST-exposure saccadic gain over both sessions and saccades (**C**). *t*-tests: ***p < 0.001.

The mean gain values measured in the PRE- and POST-exposure phases are illustrated in Figure 9. Note that the gain decreased in POST-exposure relative to PRE-exposure for both leftward and rightward saccades, consistent with the findings reported above for the gain changes during Exposure. The three-way rm-ANOVA revealed a significant main effect of the Phase factor (*F*(1, 17) = 148.558, *p* < 0.001; mean gain ± *SD*: PRE = 0.961 ± 0.043, POST = 0.862 ± 0.043), without any additional main effect or interaction (all *F* ≤ 4.043, all *p*s ≥ 0.06). Thus, in keeping with the slope of gain change results, these findings reveal that backward adaptation led to a significant after-effect in both IPSI and CONTRA sessions, without any significant difference between the two sessions.

We tested the effect of session order on this saccadic gain analysis by including the Group (IPSI-first vs. CONTRA-first) as a between-subjects factor in the rm-ANOVA. This Group factor had no significant main effect (*F*(1, 16) = 0.059, *p* = 0.811) and did not interact with any other factor (all *F* ≤ 3.322, all *p*s ≥ 0.087), except for the triple-interaction Group × Saccade direction × Phase (*F*(1, 16) = 5.042, *p* = 0.039). However, post hoc pairwise Bonferroni comparisons for this interaction showed neither significant differences between groups (all *p*s ≥ 0.226) nor between saccade directions (all *p*s ≥ 0.109), while the phase difference was significant for both saccade directions and counterbalanced groups (all *p*s ≤ 0.001).

In summary, we succeeded in inducing backward adaptation of both leftward and rightward VS in both IPSI and CONTRA sessions but did not observe any significant difference in adaptation parameters between sessions that might result from the orienting of attention.

#### Discrimination RT

**Figure 10.**
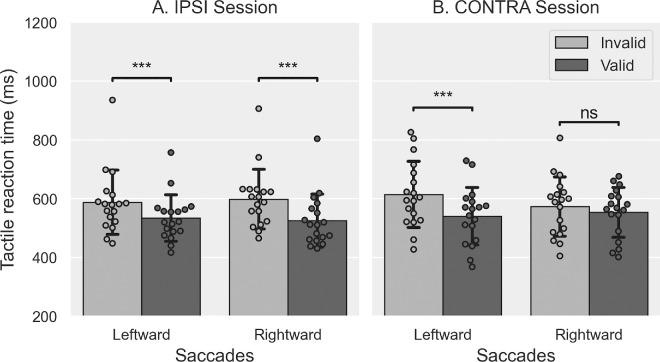
Tactile discrimination performance in Experiment 2. Mean reaction time to the tactile target (± *SD*) in leftward and rightward saccade trials for valid (dark gray) and invalid trials (light gray) of the IPSI (**A**) and the CONTRA (**B**) sessions. *t*-tests: ^ns^*p* > 0.05, ***p < 0.001.

As in Experiment 1, we analyzed the RT of the tactile discrimination responses to verify that the participants’ endogenous spatial attention was successfully oriented to the tactile cue in both the IPSI and CONTRA sessions. The mean RT values of correct responses are plotted in Figure 10. The three-way Session × Saccade direction × Validity rm-ANOVA showed a significant main effect of Validity (*F*(1, 17) = 57.101, *p* < 0.001, participants were 55.4 ms faster responding in valid vs. invalid trials), as well as of the Saccade direction × Validity (*F*(1, 17) = 5.418, *p* = 0.033) and Session × Saccade direction × Validity interactions (*F*(1, 17) = 18.239, *p* < 0.001). There were no other significant effects or interactions (all *F* ≤ 1.772, all *p*s ≥ 0.201). Bonferroni-corrected post hoc comparisons for the Session × Saccade direction × Validity interaction revealed significant differences between valid and invalid trials for all saccades and sessions (all *p*s < 0.001) except for rightward saccades in the CONTRA session (*p* = 0.098). This negative finding should be put in parallel with an additional post hoc result, namely that only for invalid trials of the CONTRA session, rightward saccades had faster RT than leftward saccades (41-ms difference, *p* = 0.004; all other RT differences between saccade directions were not significant: all *p*s ≥ 0.120).

In conclusion, spatial attention in the IPSI session could be oriented toward both sides, whereas in the CONTRA session, it was only toward the right side (i.e., for leftward saccades).

#### Correlations

**Figure 11.**
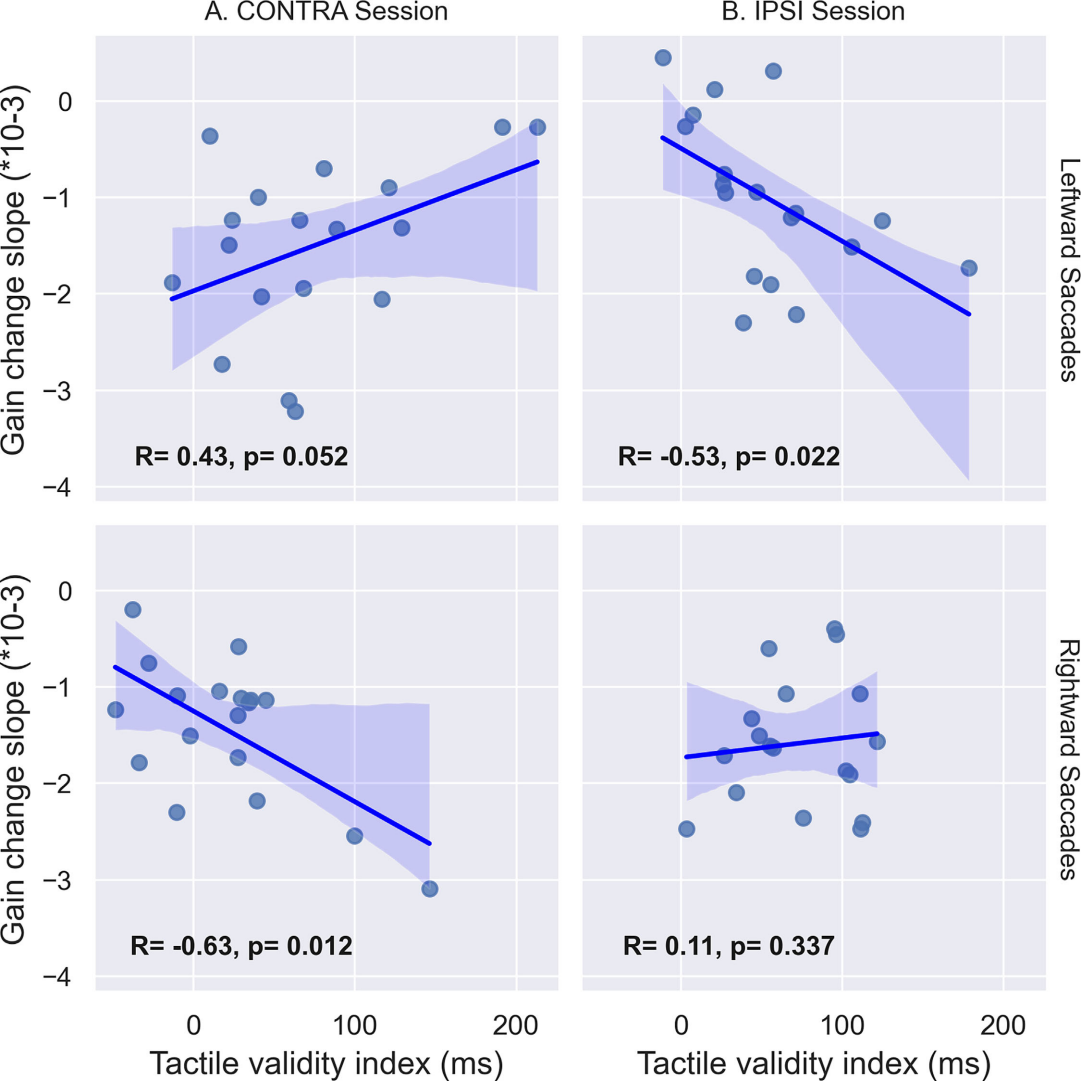
Experiment 2: correlation between the slope of gain change during time and the tactile validity index (ms) for leftward and rightward saccades in the IPSI (**B**) and the CONTRA (**A**) sessions. *R* represents Pearson's correlation value, and *p* represents the *p*-value of the one-sided Pearson correlation (corrected for multiple comparisons).

We investigated the correlations between spatial attention orienting, indexed by the tactile validity index, and the two measures of VS adaptation (gain change slope and gain change ratio). Considering first the relationship with the gain slope (Figure 11), a significant negative correlation was found in the IPSI session for leftward saccades (*R* = −0.533, *p* = 0.022) but not for rightward saccades (*R* = 0.106, *p* = 0.337), whereas in the CONTRA session, there was no significant correlation for leftward saccades (*R* = 0.426, *p* = 0.052) and a significant negative correlation for rightward saccades (*R* = −0.625, *p* = 0.012).

**Figure 12.**
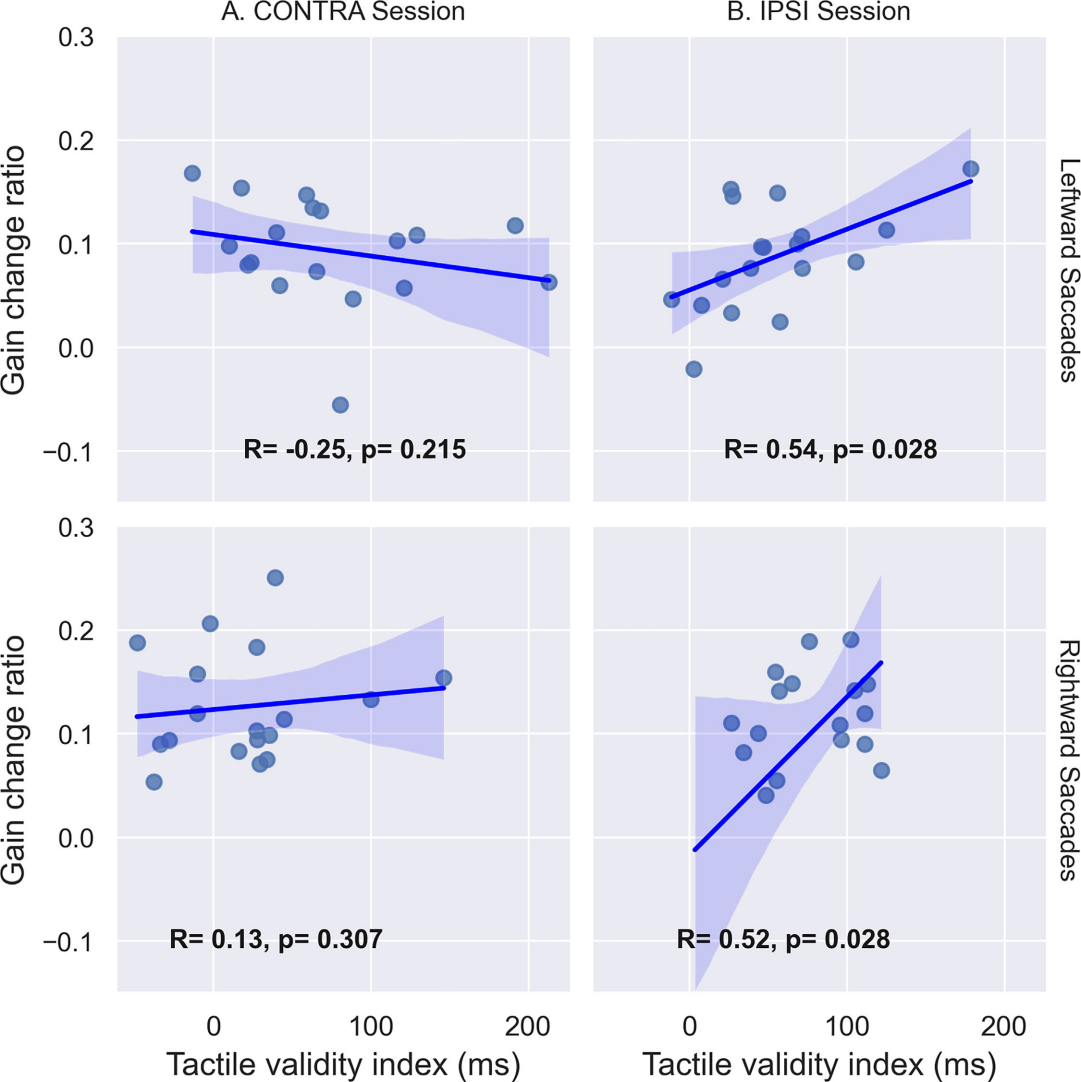
Experiment 2: correlation between the gain change ratio and the tactile validity index (ms) for left and rightward saccades in both IPSI (B) and CONTRA (A) sessions. *R* represents Pearson's correlation value, and *p* represents the *p*-value of the one-sided Pearson correlation (corrected for multiple comparisons).

**Figure 13.**
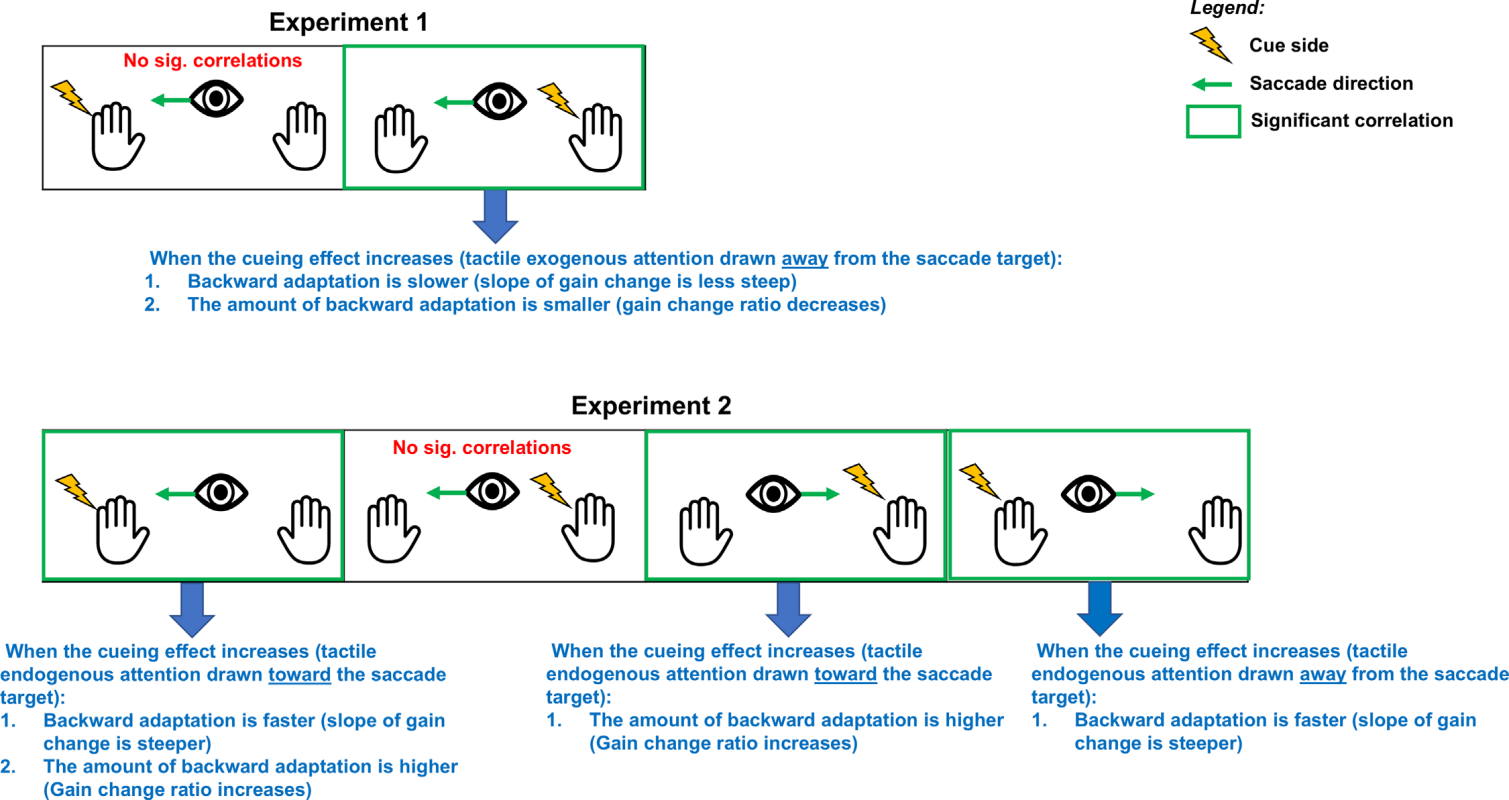
Summary of the correlation analysis from Experiment 1 (top) and Experiment 2 (bottom). The green arrow near the eye icon indicates the direction of the adapted saccade. The lightning symbol represents the location of the tactile cue (left or right hand) used to orient tactile spatial attention. Conditions in which the tactile cue and the adapted saccade are in the same hemifield correspond to the IPSI session, whereas conditions in which they are in opposite hemifields correspond to the CONTRA session. The label “No sig. correlations” indicates conditions where no significant correlations were found.

Considering now the relationship between tactile validity index and gain change ratio (Figure 12), significant positive correlations were found in the IPSI session for both leftward (*R* = 0.543, *p* = 0.028) and rightward saccades (*R* = 0.519, *p* = 0.028), whereas no significant correlations were found in the CONTRA session for either leftward saccades (*R* = −0.248, *p* = 0.215) or rightward saccades (*R* = 0.128, *p* = 0.307). The results of this analysis are further summarized in Figure 13.

### Interim discussion

In Experiment 2, we investigated the effect of the endogenous orienting of spatial attention on the adaptation of leftward and rightward VS. Similarly to Experiment 1, we were able to induce adaptation of those saccades, but statistical group analyses comparing the IPSI and CONTRA sessions did not support an effect of endogenous attention shift on VS adaptation. Since the orienting of endogenous spatial attention was not equivalent in all our conditions, we further investigated the correlation between the individually measured tactile validity index and the saccadic parameters (slope and gain change ratio). In conditions where endogenous attention was successfully oriented (as probed by the tactile validity index), correlation results indicate that the more endogenous spatial attention is oriented toward the location of the saccade's target (IPSI session), the faster the adaptation (leftward saccades) and the higher its after-effect (leftward and rightward saccades). This set of findings provides evidence for a facilitatory effect of endogenous orienting of attention on the adaptation of voluntary saccades. Yet, apparently contradicting this conclusion, the slope of gain change for rightward saccades in the CONTRA session correlated negatively with the tactile validity index, meaning that orienting endogenous attention toward the opposite location of the visual target was associated with a faster backward adaptation.

## General discussion

In this study, we investigated the impact of spatial attention on saccadic adaptation. We used tactile cueing to orient participants’ exogenous (Experiment 1) and endogenous (Experiment 2) spatial attention toward or away from the location of the saccade's target while inducing backward adaptation of reactive (Experiment 1) and voluntary saccades (Experiment 2). In both experiments, group-level analyses revealed no difference in the gain change slope (an indicator of the speed of adaptation) and the gain change ratio (an indicator of the amount of adaptation after-effect) between the spatial attention orientation conditions (toward the saccade target vs. away from it). However, tactile discrimination of validly cued targets was not uniformly speeded up across participants and conditions in comparison to invalidly cued ones, casting doubts as to the successful orienting of spatial attention in those cases. This might, therefore, hamper the comparison of adaptation parameters between sessions. Nevertheless, correlation analyses were performed to better take into account the attentional orienting capability at the individual level and revealed (as represented in Figure 13) several significant associations between this ability and the adaptation parameters (gain change slope and gain change ratio). Specifically, correlation results obtained in Experiment 1 revealed a decrease in the speed and amount of RS adaptation when exogenous attention was oriented away from the saccade's target, arguing for a facilitatory effect of exogenous attention on RS adaptation. Correlation results obtained in Experiment 2 revealed that, first, the amount of VS adaptation increases when endogenous attention is oriented toward the visual target (for both leftward and rightward saccades) and, second, the speed of leftward VS adaptation is also affected by the orienting of endogenous attention: It increases with the increasing amount of attention orientation toward the visual target; both sets of findings argue for a facilitatory effect of endogenous attention on VS adaptation. It is important to note that the sample sizes in this study were determined to provide sufficient statistical power for the planned ANOVA analyses. However, they may not have been optimal for detecting reliable correlations. Thus, the correlation findings should be interpreted with caution. Nevertheless, the data suggest that in some participants who appeared particularly sensitive to tactile cues, saccadic adaptation may have been modulated, supporting the broader conclusion that spatial exogenous and voluntary attention can influence reactive and voluntary saccade adaptation, respectively.

In their reviews, Corbetta and Shulman provided evidence that in humans, orienting spatial attention is controlled by two segregated but interacting cortical networks, the dorsal and ventral frontoparietal networks (Corbetta et al., 2008; Corbetta & Shulman, 2002). The dorsal frontoparietal network includes the IPS and the superior parietal lobule in the parietal cortex, as well as the FEF in the frontal cortex, and is thought to generate endogenous attention signals. The ventral frontoparietal network, situated in the right hemisphere, includes the rTPJ, the ventral part of the supramarginal gyrus, and the ventral frontal cortex and is thought to be responsible for stimulus-driven reorienting of attention. The ventral network interrupts the top-down effect of the dorsal network on visual areas when unexpected but relevant stimuli appear; therefore, this ventral network was frequently referred to as the “circuit breaker.” Nevertheless, many studies have shown that each frontoparietal network contributes at least to some extent to both exogenous and endogenous orienting of attention. Therefore, the psychological distinction between these two types of spatial attention orienting does not exactly match the anatomical segregation into ventral and dorsal frontoparietal networks (Chica, Bartolomeo, & Valero-Cabré, 2011; Downar, Crawley, Mikulis, & Davis, 2002; de Fockert, Rees, Frith, & Lavie, 2004; Indovina & Macaluso, 2007; Kincade, Abrams, Astafiev, Shulman, & Corbetta, 2005). Also, different studies have highlighted the presence of multimodal attentional structures in both frontoparietal networks (for review, Macaluso, 2010). For instance, in the dorsal frontoparietal network, the dorsal premotor cortex and the IPS were shown to be activated when the task involved endogenous orienting of attention, irrespective of whether participants prepared for a visual or a tactile discrimination response (Macaluso, Eimer, Frith, & Driver, 2003). Similarly, in the ventral frontoparietal network, the TPJ and the inferior frontal gyrus also show significant target-related activations irrespective of the modality of the target (tactile or visual; Macaluso, Frith, & Driver, 2002). As our study used tactile stimuli to orient spatial attention, one possibility is that the significant correlations observed between spatial attention and saccadic adaptation resulted from shared neural substrates at the level of these multimodal structures. As pointed out in the introduction, Gerardin et al. (2012) showed activation of the IPS during VS adaptation and of the rTPJ during RS adaptation. Altogether, these observations suggest that facilitation of saccadic adaptation by spatial attention might result from interactions at the level of the IPS (for endogenous attention and VS adaptation) and of the TPJ (for exogenous attention and RS adaptation). The activation of the IPS and TPJ during saccadic adaptation (Gerardin et al., 2012) has also been reported by Guillaume et al. (Guillaume, Fuller, Srimal, & Curtis, 2018) and attributed by these authors to the processing of the error signal involved in RS adaptation. Another fMRI study focusing on RS adaptation (Métais et al., 2022) also provided empirical support for the involvement of the posterior parietal cortex (PPC) in error signal processing. Furthermore, the locus of attention itself was suggested to act as an error signal leading to saccadic adaptation (Khan et al., 2014; McFadden et al., 2002). When considered in the light of such literature, our findings suggest that spatial attention may act on saccadic adaptation by improving the processing of saccadic error signals in the PPC. Another possibility would be a more indirect effect of spatial attention on the processing of error signals used for saccadic adaptation, namely by biasing the postsaccadic visual information encoded by neural activity in the visual occipital cortex. Indeed, it was shown that the dorsal network directly biases the activity of the visual occipital areas through projections from the FEF (Moore & Armstrong, 2003) and/or the IPS (Vossel, Weidner, Driver, Friston, & Fink, 2012). The ventral network would also affect the occipital area but indirectly through the dorsal network (Corbetta et al., 2008). In addition, this possibility that attention modulates saccadic adaptation at the level of the occipital cortex is also consistent with the fact that both tactile predictive (Macaluso et al., 2002; Macaluso et al., 2003) and nonpredictive (Kennett, Eimer, Spence, & Driver, 2001; Macaluso, Frith, & Driver, 2000) cues can boost the activities of the occipital visual areas contralateral to the stimulus location. Further, these neural activity modulations at the parietal and/or occipital levels, which are suggested to underlie interactions between spatial attention and saccadic adaptation, do not rule out possible modulations at the level of subcortical areas as well, such as at the SC level.

Further studies are needed to decipher whether the observed effects of spatial attention on saccadic adaptation entail facilitating the processing of postsaccadic visual information on which the error signal is built and/or directly supplying a complementary source of information for the error signal computation.

Our significant correlation results indicate an increased or decreased adaptation when attention was shifted ipsilaterally or contralaterally to the adapted target, respectively, compatible with a functional modulation of adaptation by attention. A notable exception was the slope of gain change versus tactile validity index relationship for rightward saccades when attention was oriented contralaterally to the saccade target. This could be related to the dominance of the right hemisphere in attention orienting, as detailed in the following. Indeed, orienting spatial attention toward the tactile cue contralateral to the adapted rightward saccade (i.e., toward the left hemifield) recruits attention-related areas mostly in the right hemisphere (Macaluso & Driver, 2001). In parallel, the orientation of visuospatial attention accompanying the generation of such rightward saccades implicates the activation of attentional regions not only in the left but also in the right hemisphere, due to the latter's attentional dominance (Capotosto, Babiloni, Romani, & Corbetta, 2009; Capotosto, Babiloni, Romani, & Corbetta, 2012; Corbetta & Shulman, 2002). Therefore, visuospatial attention allocated to the saccade target might be indirectly boosted by the tactile cue–induced activation of the right hemisphere, resulting in the observed negative correlation between the slope of gain change and the tactile validity index. This hypothesis is consistent with the RT analysis of corresponding trials (rightward saccades in the CONTRA session), which shows that discriminating targets in the invalid (tactile target on the right) trials was as fast as in the valid (tactile target on the left) trials. Therefore, we suggest that, while attention was intended to be oriented contralaterally to the saccade target, some unexpected facilitation also took place ipsilaterally.

Beyond its main objective, our study provided another outcome related to the different sensory modalities of attention cues and saccade targets. It has been previously shown that presenting a nonvisual stimulation before a visually triggered saccade reduces the saccade latency, specifically when the SOA between both sensory sources is less than 200 ms (Diederich & Colonius, 2008; Vidal, Desantis, & Madelain, 2020). This effect has also been shown to be stronger when the sensory stimulus is ipsilateral to the saccade target. These findings suggest a multisensory integration effect on saccade preparation, possibly taking place at the level of the SC (Diederich & Colonius, 2008; Vidal et al., 2020). The SC is indeed known to be a critical site of multisensory integration: Visual, auditory, and tactile sensory information converge in the intermediate and deep layers of the SC after being totally or partially translated into the oculocentric reference frame in which the oculomotor commands of the appropriate saccadic response toward these stimuli are generated (Groh & Sparks, 1996a; Groh & Sparks, 1996b; Jay & Sparks, 1987a; Jay & Sparks, 1987b). In Experiment 1, we observed a pattern of cue-related latency modulation compatible with multisensory integration. Indeed, RS preceded by an ipsilateral tactile cue (50 ms before the saccade target) had a shorter latency than those preceded by a contralateral cue, and in both cases, the saccade latency was reduced as compared with saccades performed in the PRE-exposure phase without a tactile cue. Thus, the findings in Experiment 1 point toward a presaccadic facilitatory effect of tactile cueing on saccade preparation, possibly resulting from a multisensory integration process at the level of the superior colliculus. In contrast, for VS in Experiment 2, we found a significant increase in the latency of saccades preceded by a cue compared to the saccades in the PRE-exposure without a cue, with no significant difference between the cue types (IPSI vs. CONTRA). The fact that the presaccadic facilitatory effect seen in Experiment 1 was not seen in Experiment 2 is not surprising, as in the latter case, the tactile cue was delivered after the saccade target onset, 380 ms before the presentation of the go-signal, and, therefore, outside the temporal window (−200 to 0 ms) critical for multisensory integration. It is also worth noting that in Experiment 2, the visual target appeared a long and variable time before the tactile cue, and this was later preceded by the go-signal by 380 ms. Because saccade preparation had likely already begun, it would be difficult to conclude whether tactile attention affected saccade latency cross-modally. However, the tactile cue reliably increased saccade latency, regardless of its position, possibly due to added uncertainty or processing demands during saccade programming.

### Limits of the study

**Figure 14.**
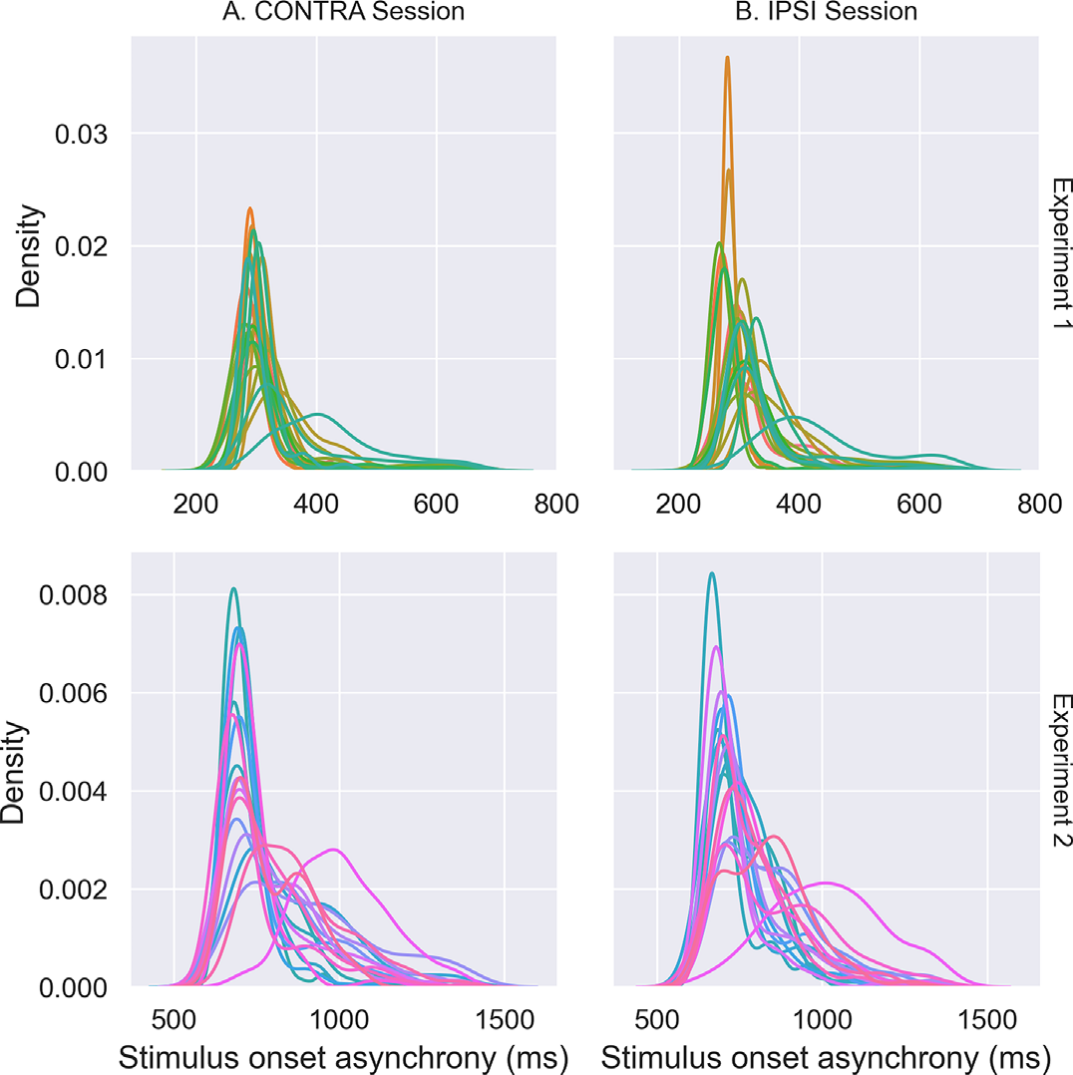
Individual distributions of SOA. The distribution densities of the stimulus onset asynchrony fitted for the different participants (different colors) are superimposed for the CONTRA (**A**) and the IPSI (**B**) sessions across both Experiments 1 and 2. These SOAs represent the time between the presentation of the tactile cue and the tactile target. It thus includes a variable time consisting of the latency and duration of the saccade, as well as a predetermined time, adding the delay from the tactile cue to the saccade target presentation and the delay from the saccade offset to the tactile target.

In this study, we leveraged the cross-modal nature of spatial attention and used tactile cues to orient spatial attention. We reasoned that visual cues would have potentially conflicted with the saccade visual targets, which would have likely hampered participants’ performance. Experiment 1 was designed to engage exogenous tactile attention with nonpredictive cues (50% valid vs. invalid trials), whereas Experiment 2 engaged endogenous tactile attention with peripheral predictive cues (80% valid vs. 20% invalid trials). While results from the discrimination task confirmed that tactile cueing facilitates (speeds up) the discrimination response for most participants and conditions, such facilitation was not observed in some conditions, also differing across experiments. While such variability was unexpected, and given the scarcity of prior research combining the orienting of tactile spatial attention with saccadic eye movements, we consider that the inclusion of a saccade between the cue and target may have added some complexity, revealing a nonuniform efficacy in spatial orienting of attention. In Experiment 1, the cueing effect in the tactile discrimination task was absent for cues on the left side, regardless of saccade direction. In contrast, in Experiment 2, the cueing effect was absent for cues on the left side only during rightward saccades (CONTRA session). The ineffective cueing might result from the following causes. First, using the right foot in a discrimination task has been suggested to speed up the participants’ response to targets presented ipsilaterally to the foot pedal, potentially masking any difference in RT between invalid and valid trials due to a floor effect (Lloyd, Azañón, & Poliakoff, 2010). In our study, despite spatial attention oriented as intended, this might have artificially masked the validity effect for Left-Cued trials (speeding up responses in these invalid trials), while increasing it for Right-Cued trials (speeding up responses in these valid trials), which is consistent with the pattern of results in Experiments 1 and 2. A second possibility is that, as discriminating a (even unseen) target is faster when fixating on its location (Honoré, Bourdeaud'hui, & Sparrow, 1989), our participants provided faster discrimination responses when fixating the unseen target location (valid trials of the IPSI session and invalid trials of the CONTRA session). However, while this difference was observed in the second experiment for rightward saccades, no such effect was observed for leftward saccades in this experiment or for both saccade types in the first experiment. A third explanation is that, in our experiments, the requirement of executing a saccade between cue and target likely introduced further variability in attentional orienting. Indeed, as saccade latencies are known to fluctuate naturally across trials, even within the same participant, the time between the presentation of the tactile cue and the tactile target also varied between trials. Nevertheless, while implementing and piloting the protocol, we took into consideration the median latency value reported in the literature for both reactive (around 180 ms) and voluntary (around 300 ms) saccades, as well as their duration (50 ms), ensuring that in both experiments, the mean delays between the cue and the target were adequate for a facilitatory cueing effect. Indeed, as depicted in Figure 14, most of our participants’ SOAs (the time between the presentation of the tactile cue and the tactile target) fell between 150 and 400 ms in Experiment 1 and between 500 and 1,200 ms in Experiment 2. According to the literature (Chica et al., 2007; Plax, 2021; Spence et al., 1998; Spence, Pavani, & Driver, 2000; Spence & McGlone, 2001), these SOA ranges were adequate to successfully orient spatial attention toward the tactile cue and, through a cross-modal effect, to facilitate the visual processing of saccade targets.

A limitation of the present study is that, although we relied on the well-established cross-modal link in spatial attention—namely, that orienting attention in the tactile modality can enhance visual processing at the cued location—we did not include a direct measure of visuospatial attention. Thus, our conclusions regarding the influence of the orienting of spatial attention on saccadic adaptation should be considered with this limitation in mind. Future studies specifically assessing visuospatial attention shifts will be needed to confirm this preliminary evidence.

In conclusion, by leveraging the cross-modal link of attention between touch and vision, this study reports initial evidence that both exogenous and endogenous shifts of spatial attention affect the adaptation of reactive and voluntary saccades, respectively. We suggest that these effects might be related to spatial attention acting on the processing of error signals underlying saccadic adaptation.
